# RUNX1 positively regulates the ErbB2/HER2 signaling pathway through modulating SOS1 expression in gastric cancer cells

**DOI:** 10.1038/s41598-018-24969-w

**Published:** 2018-04-23

**Authors:** Yoshihide Mitsuda, Ken Morita, Gengo Kashiwazaki, Junichi Taniguchi, Toshikazu Bando, Moeka Obara, Masahiro Hirata, Tatsuki R. Kataoka, Manabu Muto, Yasufumi Kaneda, Tatsutoshi Nakahata, Pu Paul Liu, Souichi Adachi, Hiroshi Sugiyama, Yasuhiko Kamikubo

**Affiliations:** 1 Department of Human Health Sciences, Graduate School of Medicine, Kyoto University, Sakyo-ku, Kyoto, 606-8507 Japan; 2 Department of Chemistry, Graduate School of Science, Kyoto University, Sakyo-ku, Kyoto, 606-8502 Japan; 3 Department of Diagnostic Pathology, Kyoto University Hospital, Sakyo-ku, Kyoto, 606-8507 Japan; 4 Department of Therapeutic Oncology, Graduate School of Medicine, Kyoto University, Sakyo-ku, Kyoto, 606-8507 Japan; 5 Division of Gene Therapy Science, Department of Genome Biology, Graduate School of Medicine, Osaka University, Osaka, 565-0871 Japan; 6 Drug Discovery Technology Development Office, Center for iPS cell research and application (CiRA), Kyoto University, Sakyo-ku, Kyoto, 606-8507 Japan; 7 Oncogenesis and Development Section, National Human Genome Research Institute, National Institutes of Health, Bethesda, MD 20892 USA; 8 Department of Pediatrics, Graduate School of Medicine, Kyoto University, Sakyo-ku, Kyoto, 606-8507 Japan

## Abstract

The dual function of runt-related transcriptional factor 1 (RUNX1) as an oncogene or oncosuppressor has been extensively studied in various malignancies, yet its role in gastric cancer remains elusive. Up-regulation of the ErbB2/HER2 signaling pathway is frequently-encountered in gastric cancer and contributes to the maintenance of these cancer cells. This signaling cascade is partly mediated by son of sevenless homolog (SOS) family, which function as adaptor proteins in the RTK cascades. Herein we report that RUNX1 regulates the ErbB2/HER2 signaling pathway in gastric cancer cells through transactivating SOS1 expression, rendering itself an ideal target in anti-tumor strategy toward this cancer. Mechanistically, RUNX1 interacts with the RUNX1 binding DNA sequence located in *SOS1* promoter and positively regulates it. Knockdown of *RUNX1* led to the decreased expression of SOS1 as well as dephosphorylation of ErbB2/HER2, subsequently suppressed the proliferation of gastric cancer cells. We also found that our novel RUNX inhibitor (Chb-M’) consistently led to the deactivation of the ErbB2/HER2 signaling pathway and was effective against several gastric cancer cell lines. Taken together, our work identified a novel interaction of RUNX1 and the ErbB2/HER2 signaling pathway in gastric cancer, which can potentially be exploited in the management of this malignancy.

## Introduction

Gastric cancer is the fourth most commonly diagnosed cancer and the second most common cause of cancer-related deaths in the world^1,2^. About 8–17% of gastric cancer patients have *HER2* gene amplification, which is associated with poor prognosis^3^. HER2 is a well-established therapeutic target in gastric cancer and patients with *HER2*-overexpressing tumors have benefit from HER2-targeted therapy with trastuzumab, pertuzumab and lapatinib^4–7^. However, most patients with advanced *HER2* gene-amplified gastric cancer eventually relapse after treatment, suggesting that tumors acquire or intrinsically possess mechanisms to escape from HER2 inhibition, necessitating other strategies to control HER2-positive gastric cancer^8,9^. Receptor tyrosine kinases (RTKs) have previously been shown to regulate the Ras/MAPK pathway by stimulating a transient interaction between the receptor and the guanine nucleotide exchange SOS family proteins^10–14^. Upon stimulation of RTKs, SOS proteins act as adaptors to augment the Ras/MAPK signaling, thereby thought to significantly contribute to the proliferation of the cells. Indeed, increased expression of *SOS1* has been identified in several types of cancers^15–17^.

RUNX1, a member of RUNX family transcription factors (RUNX1, RUNX2 and RUNX3), is an essential transcription factor mediating diverse functions in mammalian cells and modulates the transcription of its target genes through recognizing the core consensus DNA binding sequences, classically 5′-TGTGGT-3′^18–20^. We have previously reported that RUNX1 is strongly required for the maintenance and progression of acute myeloid leukemia (AML) and RUNX cluster inhibition would be a novel strategy to control AML^21–24^. We have also discovered that PI polyamides which could specifically recognize and bind to RUNX binding sites strongly inhibit the proliferation of various types of cancers including gastric cancer, suggesting that RUNX1 inhibition could be a legitimate therapeutic choice in the management of gastric cancer^22^. On the other hand, Boregowda *et al*. have recently demonstrated that RUNX2-deficient melanoma cells display a significant decrease in three receptor tyrosine kinases, EGFR, IGF-1R and PDGFRβ^25^. This report suggests a possible existence of undiscovered roles of RUNX transcription factors in the regulation of receptor tyrosine kinases in some types of tumor cells, which prompted us to investigate the potential interaction of RUNX1 and receptor tyrosine kinases in the gastric cancer cells.

In this report, we show evidence that RUNX1 plays a key role in the maintenance of gastric cancer cells by regulating the ErbB2/HER2 signaling pathway. Our findings suggest that RUNX1 inhibition therapy potentially constitutes a novel therapeutic strategy toward HER2-positive gastric cancer.

## Results

### RUNX1 confers cell proliferative advantage *via* ErbB2/HER2 signaling

**Figure 1:**
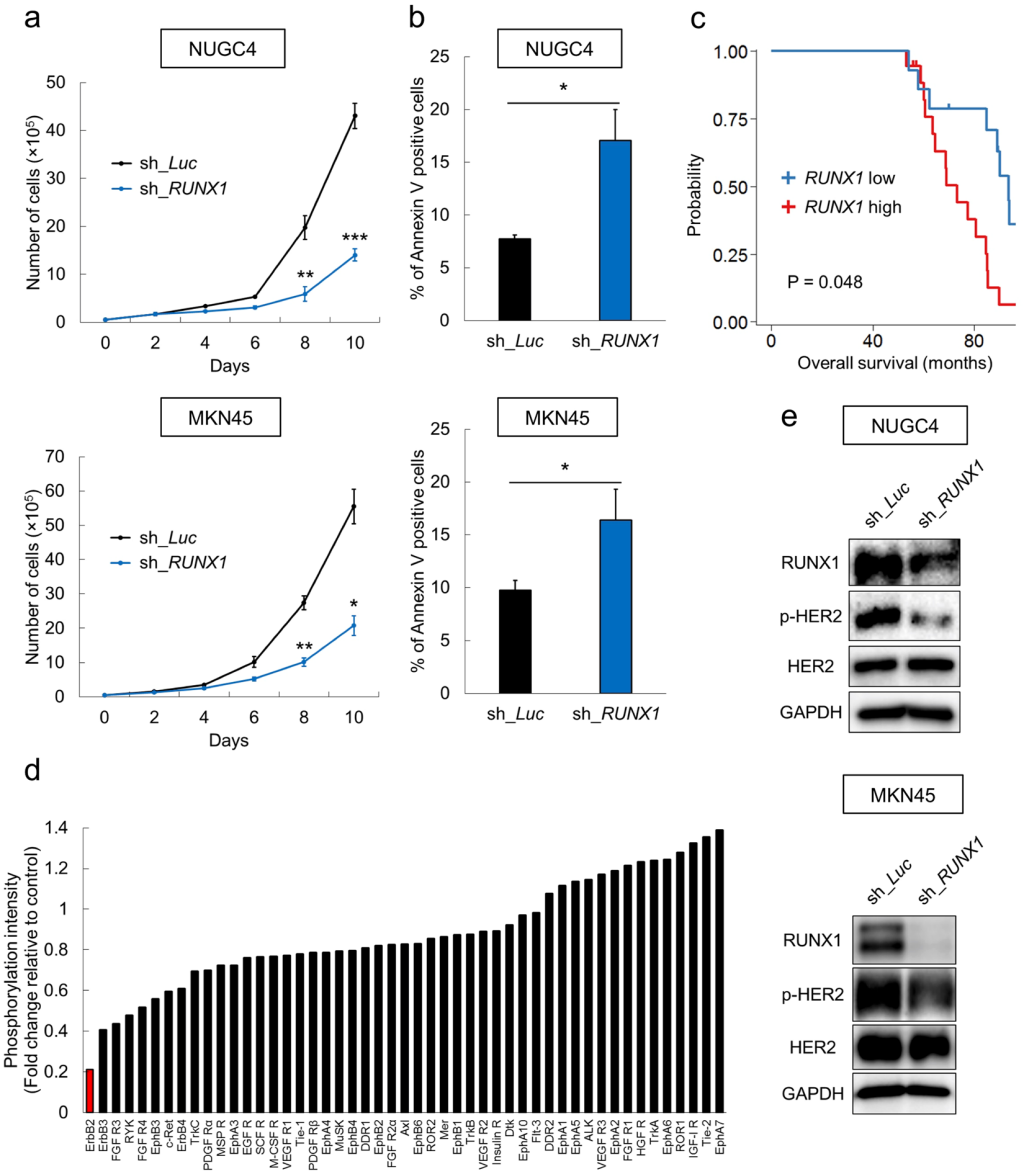
RUNX1 confers cell proliferative advantage *via* ErbB2/HER2 signaling. (**a**) Growth curves of NUGC4 and MKN45 cells lentivirally-transduced with control (sh_*Luc*) or with *RUNX1* shRNA (sh_*RUNX1*) in the presence of 3 μM doxycycline (n = 5). (**b**) Apoptotic cell death induced by *RUNX1* silencing. Non-depleted and *RUNX1*-depleted NUGC4 and MKN45 cells were cultured in the presence of 3 μM doxycycline. Forty-eight hours after treatment, cells were harvested and apoptotic cells (Annexin V^+^) were scored by flow cytometric analysis (n = 5). (**c**) *RUNX1* expressions were associated with overall survival in the gastric cancer patients. The number of subjects in *RUNX1* high (top 10%) and low (bottom 10%) were n = 30 and n = 30. P = 0.048 by log-rank test. Data was retrieved from Gene Expression Omnibus (GEO), accession number; GSE62254. (**d**) Relative densitometric quantification of phospho-RTK array spots in *RUNX1*-depleted MKN45 cells compared to the control. Cells were treated with 3 μM doxycycline for 48 hours, then the cells were lysed for the phospho-RTK array. Each receptor was spotted in duplicates (see Supplementary Fig. 1c for the immunoblot image). (**e**) Dephosphorylation of HER2 in *RUNX1*-silenced NUGC4 and MKN45 cells. Non-depleted and *RUNX1*-depleted cells were treated as in (b). Cell lysates were analyzed by immunoblotting with the indicated antibodies. Data are mean ± SEM values. *P < 0.05, **P < 0.01, by two-tailed Student’s t-test.

We first investigated whether depletion of *RUNX1* could have an anti-tumor effect on gastric cancer cells by using the tetracycline-inducible short hairpin RNA-mediated *RUNX1* knockdown system. As shown in Fig. 1a,b, silencing of *RUNX1* inhibited the growth of NUGC4 and MKN45 cells and induced apoptotic cell death. NUGC4 cells were originally established from a metastatic paragastric lymph node of a 35-year-old female with signet ring cell gastric adenocarcinoma and have significantly-upregulated expression of HER2. MKN45 cells were derived from a poorly-differentiated adenocarcinoma of the stomach of a 62-year-old woman and are known for MET amplification. These results prompted us to explore the association of *RUNX1* expression levels and prognosis among gastric cancer patients. We thus examined it in a gastric cancer cohort from Gene Expression Omnibus (GEO) dataset (GSE62254, n = 300). We divided the patients into the following groups; *RUNX1* high (top 10% of all patients; n = 30) and *RUNX1* low (bottom 10% of all patients; n = 30) according to their *RUNX1* expressions and compared their clinical outcomes. Intriguingly, as shown in Fig. 1c, we found that *RUNX1* high-expressing gastric cancer patients exhibited significantly worse clinical outcomes than *RUNX1* low-expressing patients. To investigate the underlying molecular mechanisms of RUNX1 in the tumorigenesis of gastric cancer cells, we next conducted human phospho-RTK array in MKN45 cells transduced with shRNA targeting *RUNX1* or control *luciferase* and screened the relative phosphorylation levels of 49 RTKs in these samples. Interestingly, as shown in Fig. 1d and Supplementary Fig. 1a, the level of the phosphorylation of ErbB2/HER2 was specifically and most profoundly decreased upon *RUNX1* knockdown. To confirm our finding, we performed immunoblot experiment and validated that *RUNX1*-silencing indeed dephosphorylated ErbB2/HER2 in the gastric cancer cells (Fig. 1e). These data collectively indicate that RUNX1 functions as an oncogene in gastric cancers possibly through enhancing the activity of the ErbB2/HER2 signaling pathway.

### SOS1 is up-regulated in *RUNX1* high-expressing gastric cancers

**Figure 2:**
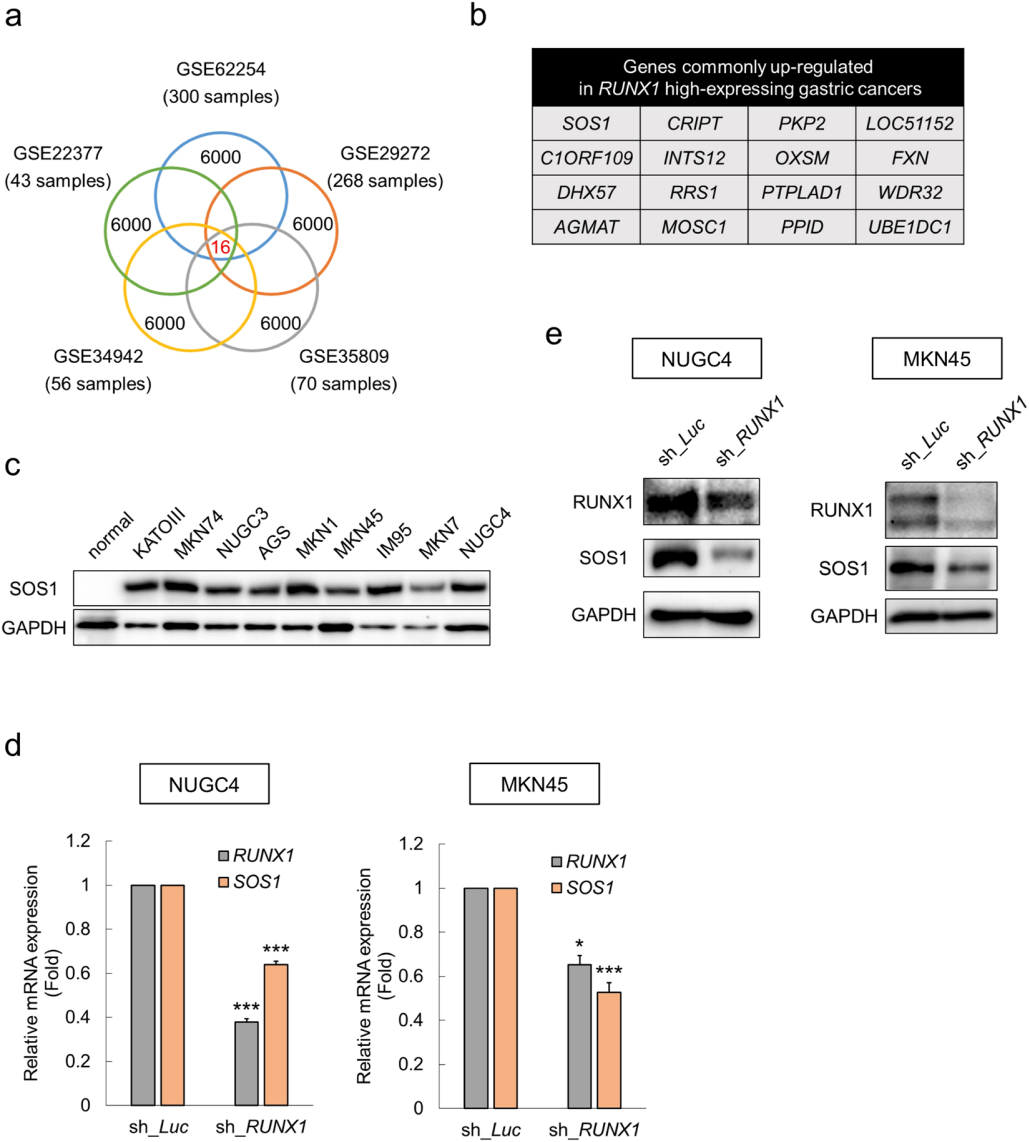
SOS1 is up-regulated in *RUNX1* high-expressing gastric cancers. (**a**,**b**) Identification of genes under the control of RUNX1 transcription factor. Sixteen genes were commonly up-regulated in 5 independent gastric cancer patients with a higher *RUNX1* expression (GSE62254, GSE29272, GSE35809, GSE34942 and GSE22377). (**c**) Up-regulation of SOS1 in the gastric cancer cell lines relative to the normal stomach tissue (total protein lysate of the whole stomach tissue: Catalog #: P1234248, Lot #: A208104, BioChain). Cell lysates were processed for immunoblotting with the indicated antibodies. (**d**) Down-regulation of *SOS1* expression upon *RUNX1* inhibition. Non-depleted and *RUNX1*-depleted NUGC4 and MKN45 cells were incubated with 3 μM doxycycline. Twenty-four hours after treatment, total RNA was prepared and analyzed by real-time RT-PCR. Values were normalized to that of control vector-transduced cells (n = 3). (**e**) Down-regulation of SOS1 expression upon *RUNX1* inhibition in NUGC4 and MKN45 cells. Non-depleted and *RUNX1*-depleted cells were treated as in (**d**). Forty-eight hours after treatment, cell lysates were prepared and subjected to immunoblotting. Data are mean ± SEM values. *P < 0.05, ***P < 0.001, by two-tailed Student’s t-test.

To explore the underlying molecular mechanisms of RUNX1-mediated regulation of the ErbB2/HER2 signaling pathway, we first extracted the top one-third genes (6,000 genes) that are highly-expressed in the *RUNX1* high-expressing gastric cancer patients in 5 independent previously-published microarray data sets (GSE62254, GSE29272, GSE35809, GSE34942 and GSE22377). As shown in Fig. 2a,b, we found 16 genes which were consistently highly-expressed in the *RUNX1* high-expressing primary gastric cancer cells. Among them, we found *SOS1*, a known adaptor protein associated with RTKs. Intriguingly, we found that SOS1 protein was also highly-expressed in the gastric cancer cell lines relative to the normal gastric tissue (Fig. 2c). Moreover, knockdown of *RUNX1* in NUGC4 and MKN45 cells significantly suppressed the expression of SOS1 both at the protein and mRNA levels (Fig. 2d,e). These data led us to hypothesize that RUNX1 possibly controls the ErbB2/HER2 signaling cascade through modulating the expression of SOS1 in gastric cancer cells.

### Indispensable role of SOS1 in the ErbB2/HER2 signaling pathway

**Figure 3:**
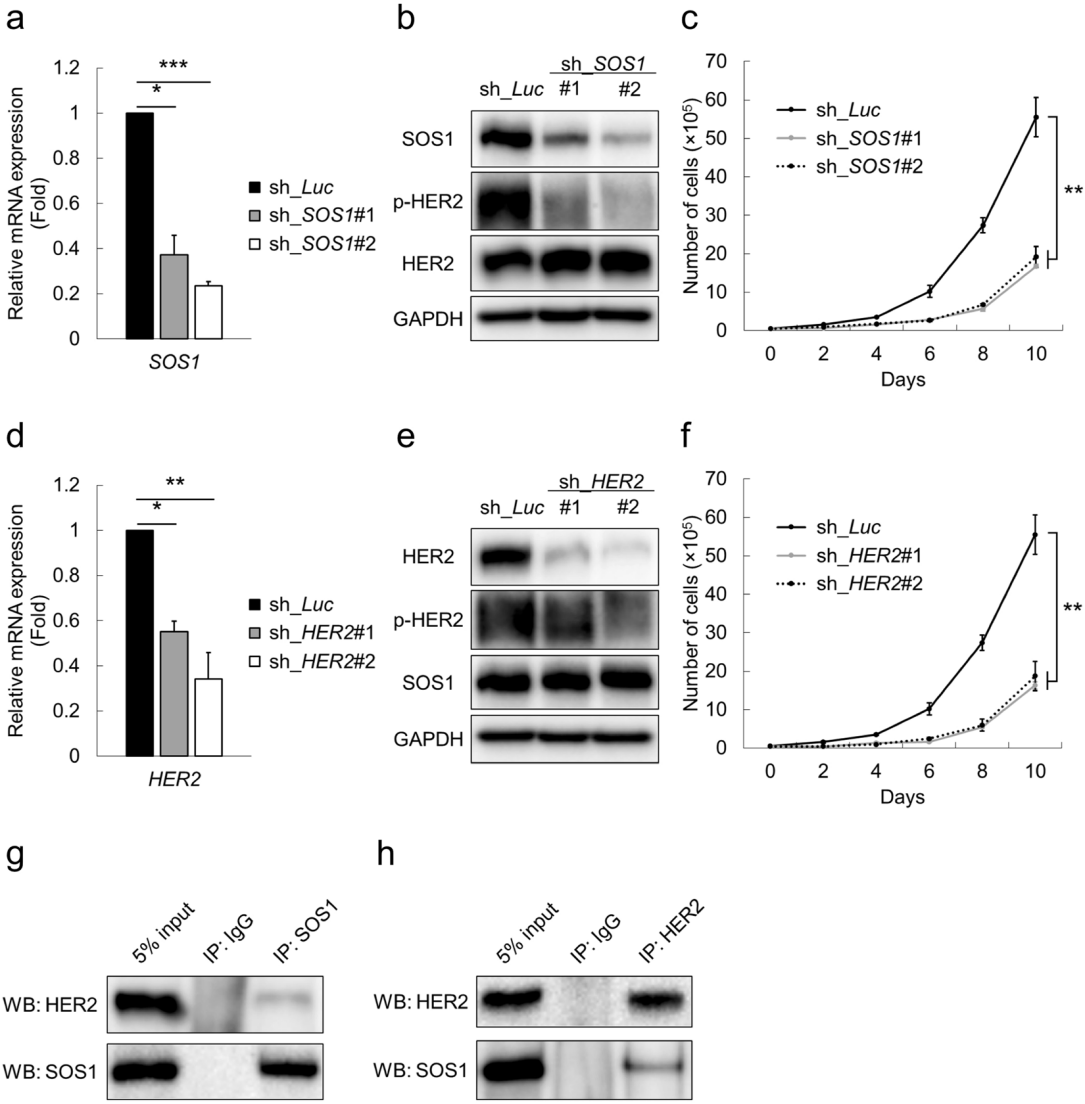
Indispensable role of SOS1 in the ErbB2/HER2 signaling cascade. (**a**) Efficacy of shRNAs targeting *SOS1*. MKN45 cells were transduced with control (sh_*Luc*) or with *SOS1* shRNAs (sh_*SOS1*#1 and #2) and cultured in the presence of 3 μM doxycycline. Twenty-four hours after treatment, total RNA was prepared and analyzed by real-time RT-PCR. Values were normalized to that of control vector-transduced cells (n = 3). (**b**) *SOS1*-depletion-mediated dephosphorylation of HER2. Non-depleted and *SOS1*-depleted MKN45 cells were treated as in (**a**). Forty-eight hours after treatment, cell lysates were processed for immunoblotting. (**c**) Growth curves of MKN45 cells transduced with control (sh_*Luc*) or with *SOS1* shRNAs (sh_*SOS1*#1 and #2) in the presence of 3 μM doxycycline (n = 5). (**d**) Efficacy of shRNAs targeting *HER2*. MKN45 cells were transduced with control (sh_*Luc*) or with *HER2* shRNAs (sh_*HER2*#1 and #2) and cultured in the presence of 3 μM doxycycline. Twenty-four hours after treatment, total RNA was prepared and analyzed by real-time RT-PCR. Values were normalized to that of control vector-transduced cells (n = 3). (**e**) *HER2*-depletion-mediated dephosphorylation of HER2. Non-depleted and *HER2*-depleted MKN45 cells were treated as in (**d**). Forty-eight hours after treatment, cell lysates were processed for immunoblotting. (**f**) Growth curves of MKN45 cells transduced with control (sh_*Luc*) or with *HER2* shRNAs (sh_*HER2*#1 and #2) in the presence of 3 μM doxycycline (n = 5). (**g**,**h**) Immunoprecipitation assay showed SOS1-HER2 complex in MKN45 cells. Data are mean ± SEM values. *P < 0.05, **P < 0.01, ***P < 0.001, by two-tailed Student’s t-test.

Although SOS family proteins have been shown to take pivotal roles in the RTKs signaling cascades^10,11^ and higher expressions of *SOS1* in solid tumors relative to their normal counterpart tissues have been repeatedly reported^15–17^, its role in gastric cancer cells has remained elusive so far. We thus investigated the oncogenic role of SOS1 in gastric cancer cells. For this purpose, we prepared shRNAs specifically targeting *SOS1* (Fig. 3a) and examined their effects on the maintenance of gastric cancer cells. As shown in Fig. 3b, HER2 was significantly dephosphorylated in *SOS1*-depleted MKN45 cells relative to the control. In addition, *SOS1* knockdown slowed down the proliferation speed of MKN45 cells (Fig. 3c). Together with our finding that *HER2*-depletion indeed decelerates the growth rate of MKN45 cells (Fig. 3d–f), these data collectively underpin the importance of SOS1 expression in the ErbB2/HER2 signaling cascade and in the maintenance of gastric cancer cell lines. Furthermore, as shown in Fig. 3g,h, immunoprecipitation assay proved the physiological interaction of SOS1 and HER2 in MKN45 cells, indicating that SOS1 probably functions as an adaptor protein in the RTKs signaling pathways like other SOS family proteins do^10,11^. Together with the results obtained in the *RUNX1* knockdown experiments, we were convinced that RUNX1 controls the ErbB2/HER2 signaling cascade through modulating SOS1 expression in gastric cancer cells.

### SOS1 expression is directly transactivated by RUNX1

**Figure 4:**
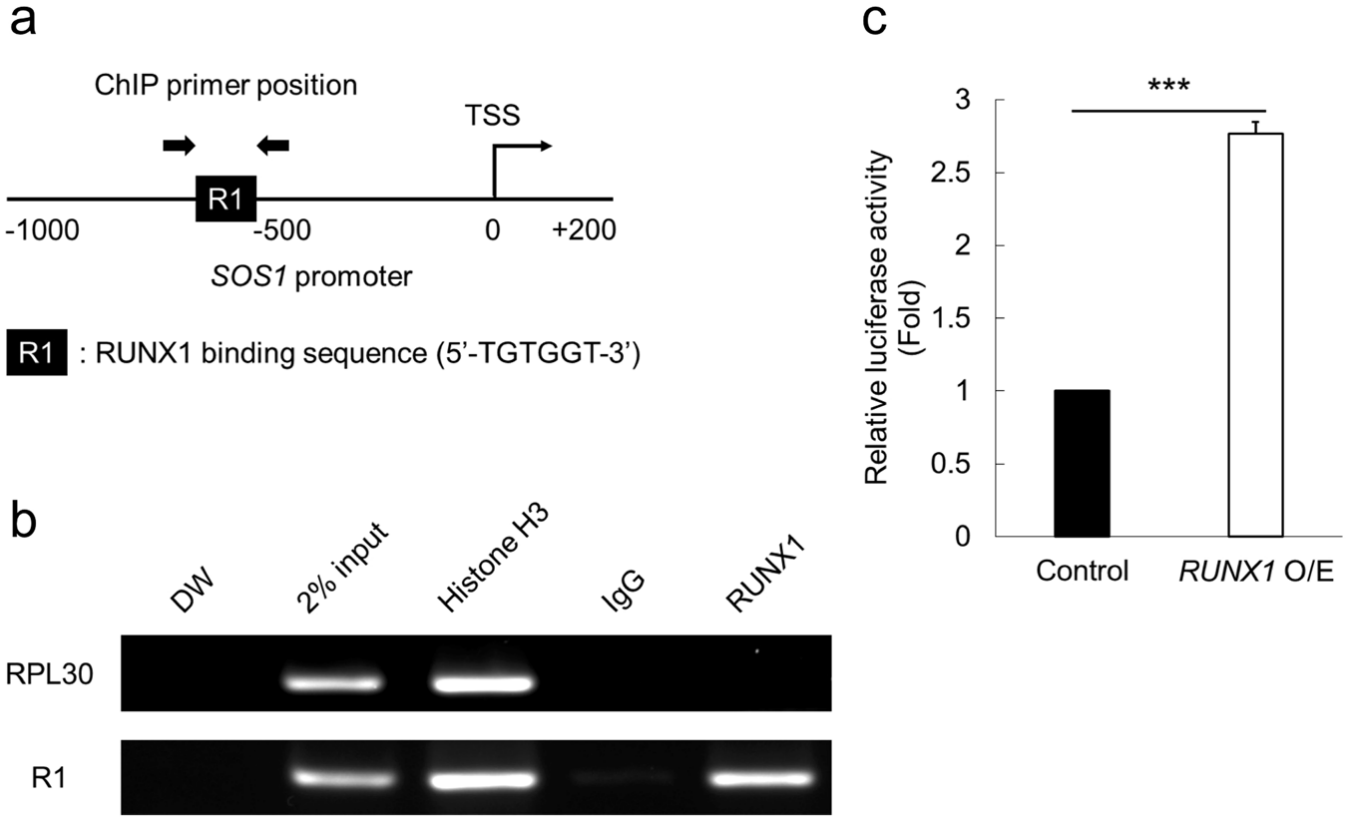
RUNX1 directly transactivates SOS1 expression. (**a**) Proximal regulatory region (−1000 bp to +200 bp relative to TSS) of *SOS1*. (**b**) Result of the ChIP analysis in MKN45 cells using anti-RUNX1 antibody, an isotype-matched control IgG and anti-Histone H3 antibody. ChIP products were amplified by PCR to determine the abundance of the indicated amplicons. (**c**) Luciferase reporter assay with *SOS1* promoter. HEK293T cells were stably-transduced with the lentivirus expressing *RUNX1* (*RUNX1* O/E) or control, together with the reporter vector expressing luciferase gene under *SOS1* promoter. Cells were incubated with 3 μM doxycycline for 48 hours, then the luciferase activity was monitored by a luminometer. Result was normalized to that of the control sample (n = 3). Data are mean ± SEM values. ***P < 0.001, by two-tailed Student’s t-test.

**Figure 5:**
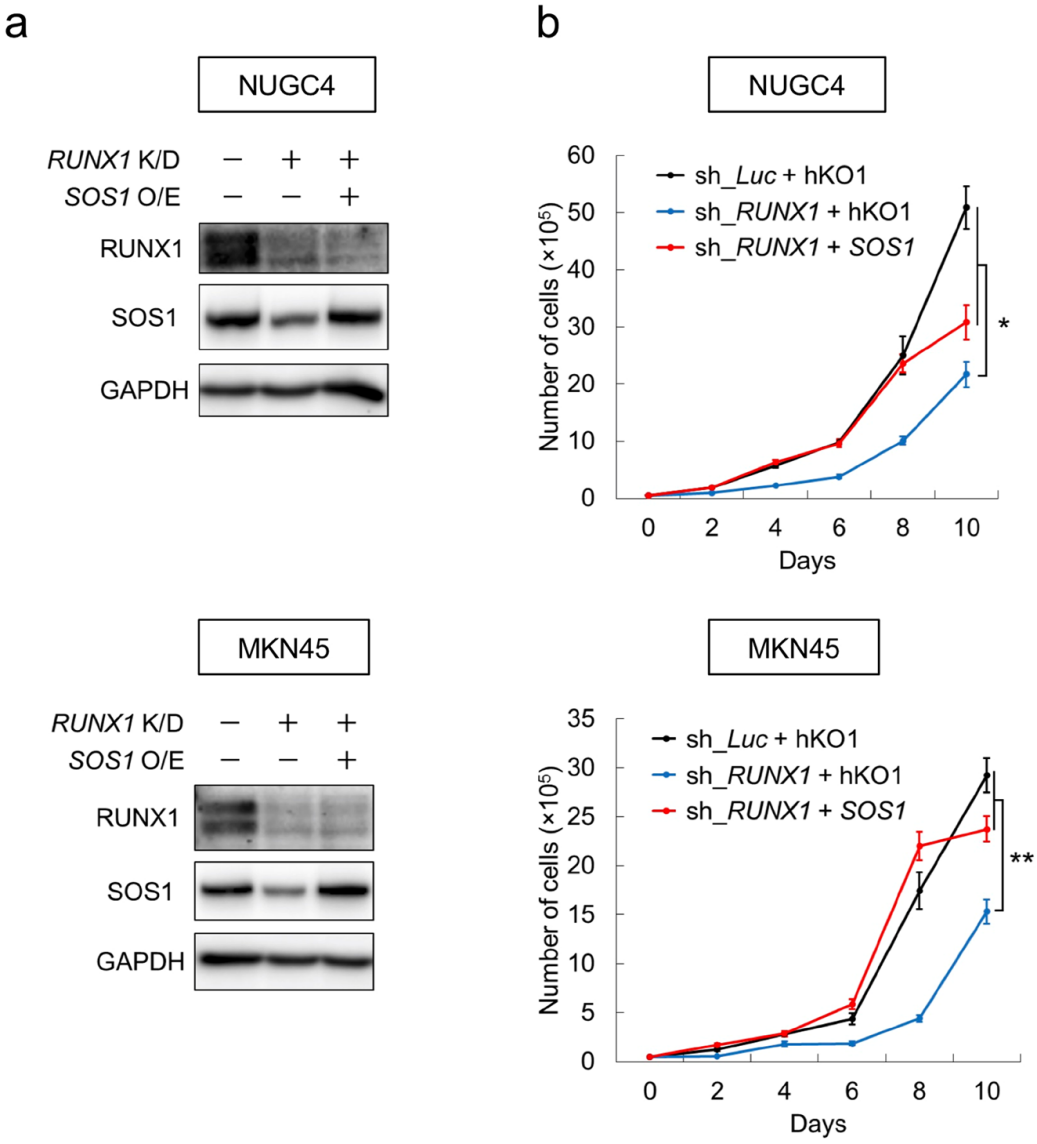
Anti-tumor effect of RUNX1 inhibition is SOS1-dependent. (**a**) Immunoblotting of RUNX1, SOS1 and GAPDH in non-depleted and *RUNX1*-depleted (*RUNX1* K/D) NUGC4 and MKN45 cells transduced with or without lentivirus expressing *SOS1* (*SOS1* O/E). Cells were treated with 3 μM doxycycline for 48 hours, then lysed for immunoblotting. (**b**) Restoring *SOS1* expression in *RUNX1*-depleted NUGC4 and MKN45 cells reverts *RUNX1*-depletion-mediated growth inhibition. The indicated cells were cultured in the presence of 3 μM doxycycline (n = 5). Data are mean ± SEM values. **P < 0.01, by two-tailed Student’s t-test.

To further elucidate how RUNX1 controls the expression of SOS1, we next performed chromatin immunoprecipitation (ChIP) assay using the promoter region of *SOS1*. As shown in Fig. 4a,b, we observed the actual binding of RUNX1 in this region. Luciferase reporter assay with *SOS1* promoter (−1000 bp to +200 bp of transcription start site (TSS) of *SOS1*) showed statistically-significant elevation of reporter signals upon additive expression of *RUNX1* (Fig. 4c). These results indicate that RUNX1 directly interacts with *SOS1* promoter and positively regulates it. As shown in Fig. 5a,b, restoring the expression of *SOS1* in *RUNX1*-knocked down NUGC4 or MKN45 cells consistently reverted the *RUNX1*-depletion-mediated growth inhibition. Taken together, these data collectively suggested that RUNX1 positively regulates the ErbB2/HER2 signaling pathway through directly transactivating SOS1 expression in the gastric cancer cells.

### RUNX inhibitor Chb-M’ induces dose-dependent apoptosis in gastric cancer cells

**Figure 6:**
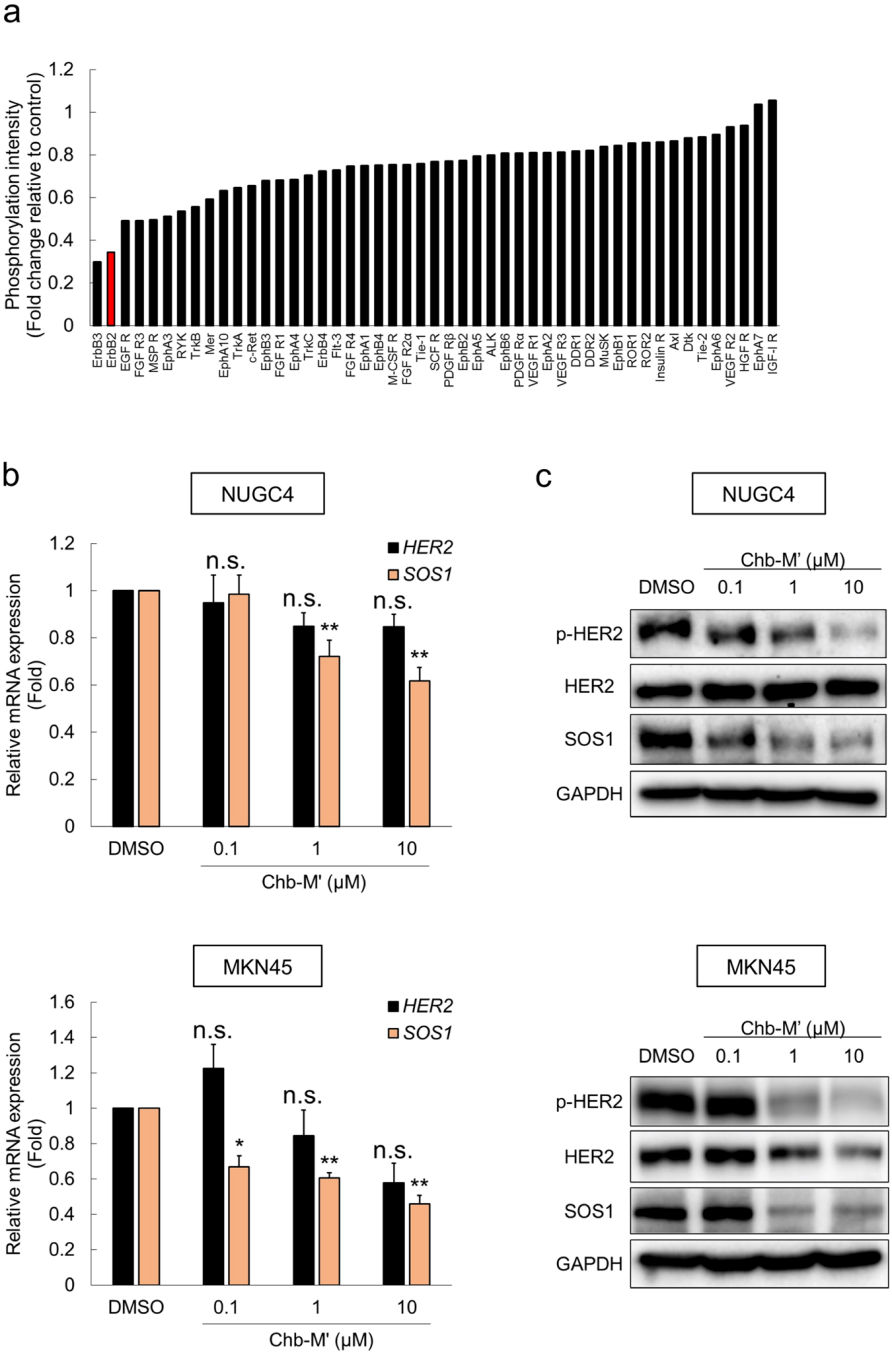
RUNX inhibitor Chb-M’ down-regulates ErbB2/HER2 pathway through attenuating SOS1 expression in gastric cancer cells. (**a**) Relative densitometric quantification of phospho-RTK array spots in Chb-M’-treated MKN45 cells compared to the control. Cells were treated with DMSO or 1 μM Chb-M’ for 48 hours, then the cells were lysed for the phospho-RTK array. Each receptor was spotted in duplicates (see Supplementary Fig. 4a for the immunoblot image). (**b**) Down-regulation of *SOS1* expression in Chb-M’-treated NUGC4 and MKN45 cells. Cells were treated with the indicated concentrations of Chb-M’. Six hours after treatment, total RNA was prepared and analyzed by real-time RT-PCR. Values were normalized to that of DMSO-treated control cells (n = 3). (**c**) Down-regulation of SOS1 and dephosphorylation of HER2 in Chb-M’-treated NUGC4 and MKN45 cells. Cells were treated as in (**b**). Forty-eight hours after treatment, cell lysates were prepared and subjected to immunoblotting. Data are mean ± SEM values. *P < 0.05, **P < 0.01, n.s.; not significant, by two-tailed Student’s t-test.

**Figure 7:**
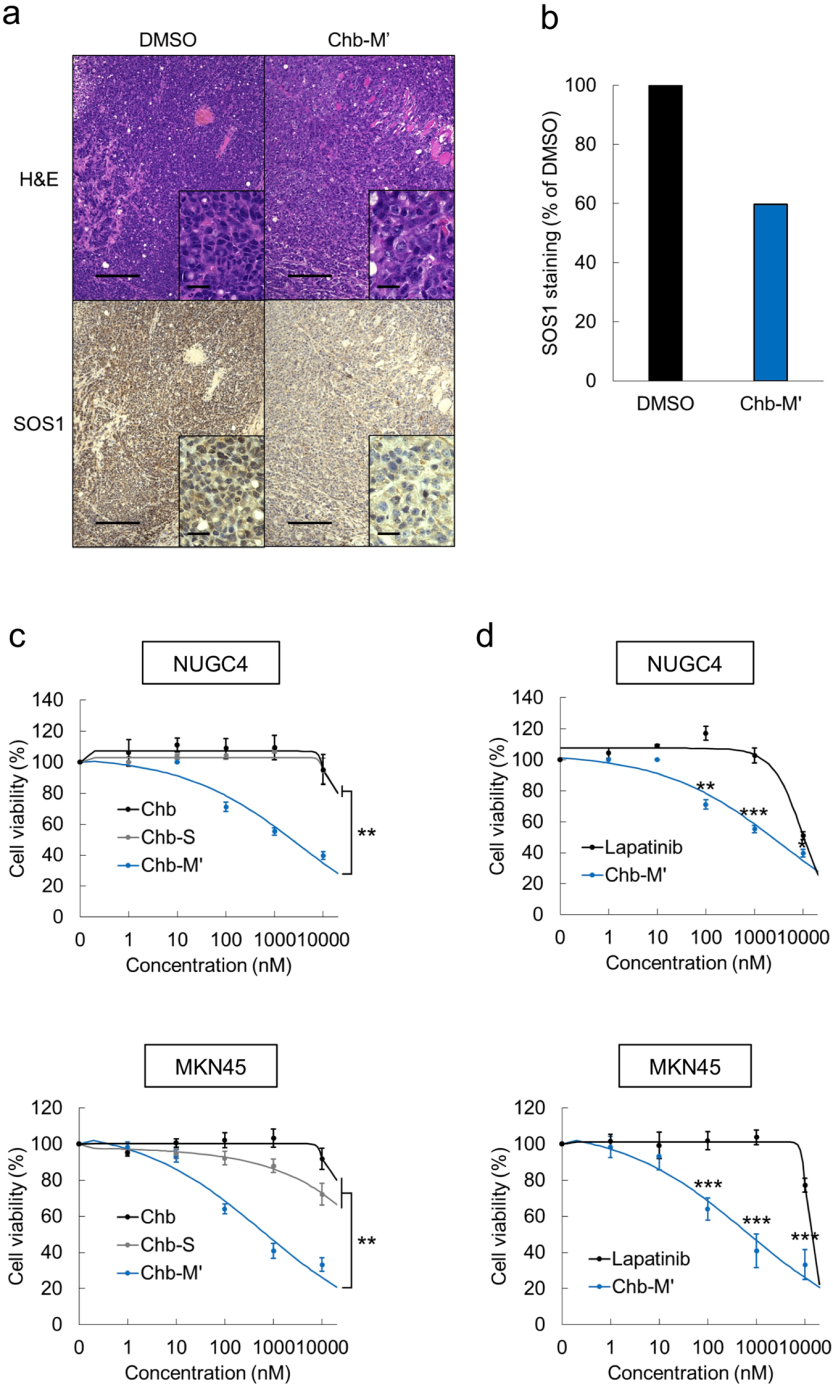
Chb-M’-mediated anti-tumor effect in gastric cancer cells. (**a**,**b**) Immunohistochemistry images of SOS1 expression in the MKN45-derived tumor treated with Chb-M’ or control DMSO. NOG mice were subcutaneously-transplanted with MKN45 cell at Day 1. Treatments either by Chb-M’ (320 μg/kg body weight, twice a week, i.v.) or equivalent amount of DMSO was continued from Day 8 until Day 21, then the mice were properly-sacrificed for tumor resection. Resected tumors were fixed with 10% formaldehyde for immunohistochemistry assay. Scale bars 200 μm for low magnification and 25 μm for high magnification (a). SOS1 staining in the MKN45-derived tumor treated with DMSO or Chb-M’ was quantified by Image J (b). (**c**) Dose-response curves of Chb, Chb-S and Chb-M’ in NUGC4 and MKN45 cells. Cells were treated with the indicated concentrations of PI polyamides or Chb. Forty-eight hours after treatment, cell viability was examined by WST assay (n = 5). (**d**) Dose-response curves of lapatinib and Chb-M’ in NUGC4 and MKN45 cells. Cells were treated as in (c). Forty-eight hours after treatment, cell viability was examined by WST assay (n = 5). Data are mean ± SEM values. *P < 0.05, **P < 0.01, ***P < 0.001, n.s.; not significant, by two-tailed Student’s t-test.

**Figure 8:**
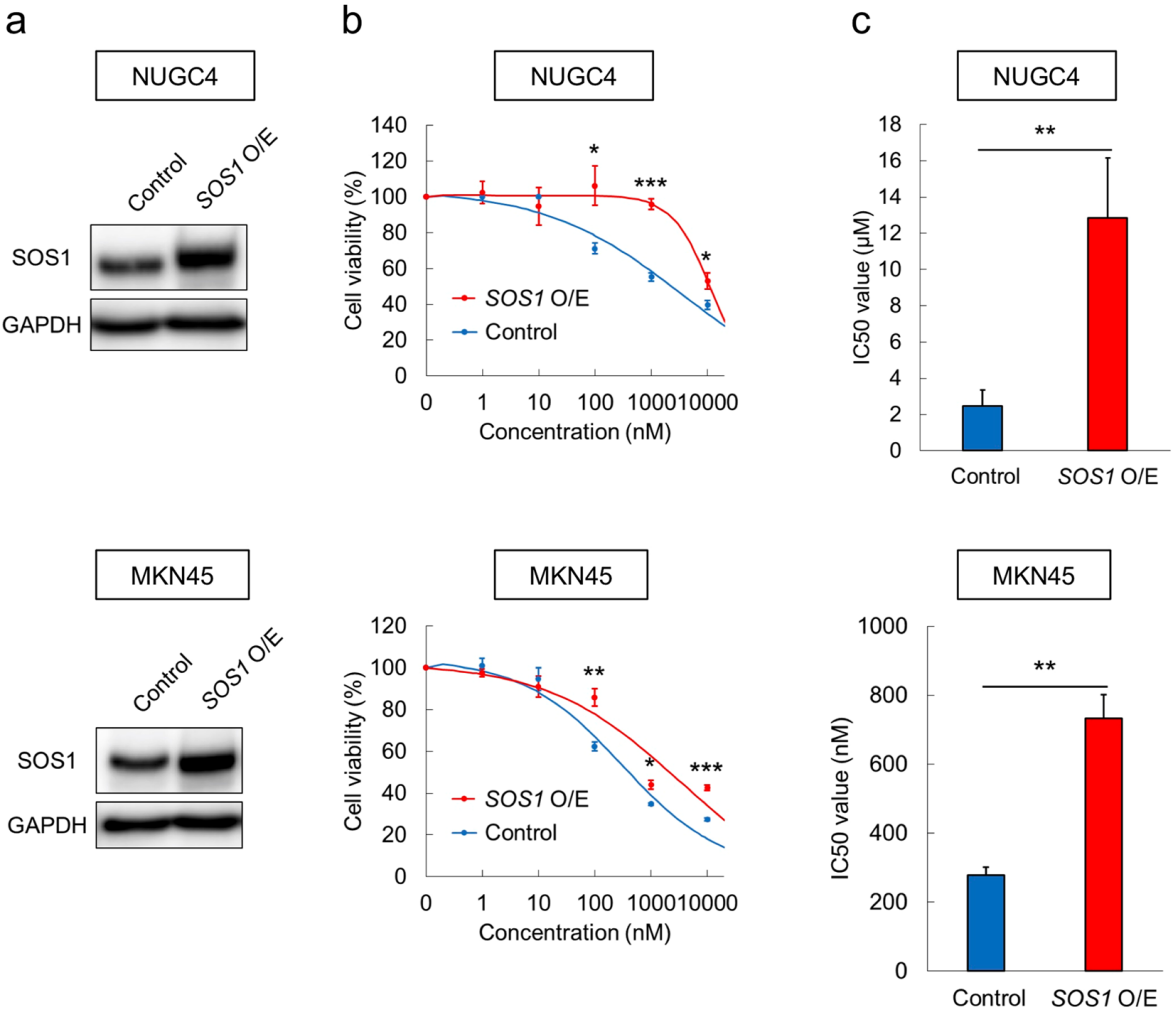
*SOS1*-overexpression confers resistance to the Chb-M’ treatment in gastric cancer cells. (**a**) Immunoblotting of SOS1 and GAPDH in NUGC4 and MKN45 cells transduced with control or *SOS1*-expressing lentivirus. Cells were treated with 3 μM doxycycline for 48 hours to induce SOS1 expression, then lysed for immunoblotting. (**b**) Dose-response curves of Chb-M’ in NUGC4 and MKN45 cells transduced either with the lentivirus expressing *SOS1* (*SOS1* O/E) or control. Cells were simultaneously treated with 3 μM doxycycline and various concentrations of Chb-M’. Forty-eight hours after treatment, cell viability was examined by WST assay (n = 5). (**c**) IC50 values of Chb-M’ in NUGC4 and MKN45 cells transduced either with the lentivirus expressing *SOS1* (*SOS1* O/E) or control. Cells were treated as in (b) (n = 5). Data are mean ± SEM values. *P < 0.05, **P < 0.01, ***P < 0.001, by two-tailed Student’s t-test.

We have previously reported a potent RUNX inhibitor Chb-M’ and its efficacy in the various types of tumor cell lines including gastric cancer^22^. In this study, we examined whether its anti-tumor potency on the gastric cancer cell lines is mediated by the RUNX1-SOS1-ErbB2/HER2 axis. As in the *RUNX1* knockdown experiments in Fig. 1, we first conducted phospho-RTK array in MKN45 gastric cancer cells treated either by Chb-M’ or control DMSO. Among 49 RTKs, the level of the phosphorylation of ErbB2/HER2 was one of the most profoundly decreased upon Chb-M’ treatment in MKN45 cells, which was consistent with the results seen in *RUNX1*-knocked down MKN45 cells (Figs 1d and 6a and Supplementary Fig. 2a). Besides, Chb-M’ suppressed the expression of SOS1 dose-dependently both at the protein and mRNA levels, and dephosphorylated HER2 in MKN45 and NUGC4 cells (Fig. 6b,c). Chb-M’-mediated down-regulation of SOS1 was also observed in the *in vivo* samples resected from the tumor, which originated from the xenotransplanted MKN45 cells in immunodeficient NOD/Shi-*scid*/IL-2Rγ^null^ (NOG) mice (Fig. 7a,b). Of note, while Chb-M’ showed significant anti-tumor effects on NUGC4 and MKN45 cells at nanomolar to low micromolar levels, chlorambucil (Chb) itself or Chb-conjugated PI polyamides that target scrambled DNA sequence (5′-WGGCCW-3′; Chb-S) were ineffective against these cells (Fig. 7c). In these cell lines, we also found the superior potency of Chb-M’ to lapatinib, a selective HER2 inhibitor currently used in the clinics (Fig. 7d). Moreover, as we have expected, *SOS1*-overexpressed NUGC4 and MKN45 cells became relatively resistant to the Chb-M’ treatment (Fig. 8a–c). These data collectively indicate that RUNX inhibition by Chb-M’ could be a novel therapeutic strategy in HER2-positive gastric cancer cases.

## Discussion

RUNX1 transcription factor plays a pivotal role in the development and maintenance of various cancers^20,22,26–29^. Although we have previously reported that the Cluster Regulation of RUNX (CROX) is a promising therapeutic strategy against various types of tumors including gastric cancer^22^, precise molecular mechanisms of RUNX-inhibition-mediated anti-tumor effect on these malignant cells have poorly been elucidated. We herein revealed that RUNX1 potentially participates in the maintenance of gastric cancer cells through enhancing the activity of the ErbB2/HER2 signaling pathway by directly transactivating SOS1 expression. We do acknowledge that RUNX transcription factors have diverse targets *in vivo* and they potentially have multiple other targets than SOS1-ErbB2/HER2 in the gastric cancer cells. In the gastric cancer cell lines tested in this study, increased activity of HER2 was seen in 4 out of 9 cell lines (44.4%). Chb-M’ treatment down-regulated the SOS1 protein expression in 7 out of 9 cell lines (77.8%) and significant anti-tumor efficacy was observed in 5 out of 9 cell lines (55.6%). All these 5 cells lines had the decreased expression of SOS1 upon Chb-M’ treatment (Supplementary Fig. 3a–c). These data suggest that SOS1 is probably one of the most important oncogenic targets of RUNX1 in the gastric cancer cells.

We do also acknowledge that MKN45 cells, one of the gastric cancer cell lines used in this study, have amplified-MET and more likely to dependent on this pathway than ErbB2/HER2 signaling for their survival. Yet, the results obtained in our shRNA-mediated *HER2* knockdown experiments suggest that this cell line is somewhat dependent on this signaling pathway, and ErbB2/HER2 signaling pathway can still be targeted to control the proliferation of this gastric cancer cell line. To further validate our results, we admit that our findings should be reexamined in a larger set of HER2-amplified gastric cancer cell lines in future studies.

Given the recent introduction of trastuzumab for the treatment of patients with advanced gastric cancer, assessment of HER2 status is now mandatory for selecting patients eligible for this treatment^6,7^. Yet, trastuzumab therapy is not curative, with only modest improvement of overall survival in the latest trials with gastric cancer patients. Though not fully elucidated, the mechanism of resistance to trastuzumab therapy seems to be based on the compensatory up-regulation of other RTKs in response to HER2 inhibition^8,9^. Though pure speculation, Chb-M’ therapy may potentially overcome the resistance of current specific HER2 inhibition therapies and provide alternative therapeutic choice for these patients, since Chb-M’ regulates the expression of SOS1, an adaptor protein that is generally-required for the RTKs to function properly. Indeed, the phosphorylation of several RTKs other than ErbB2/HER2 was simultaneously and significantly suppressed upon shRNA-mediated *RUNX1* depletion or Chb-M’ treatment in the phospho-RTK arrays (Figs 1d and 6a).

In conclusion, our work identified a novel role of RUNX1 in the ErbB2/HER2 signaling pathway in gastric cancer cells. Considering the excellent *in vivo* tolerability and strong anti-tumor efficacy of Chb-M’ in mice^22^, clinical trials of Chb-M’ in gastric cancer patients are highly-awaited to further verify the anti-tumor potency of RUNX inhibition strategy toward gastric cancers.

## Methods

### Cell lines

KATOIII, MKN74, NUGC3, MKN1, IM95 and NUGC4 cells were purchased from JCRB Cell Bank, Japan. AGS, MKN45 and MKN7 cells were kindly gifted by Dr. M. Muto (Kyoto University, Japan). Cells were maintained in Roswell Park Memorial Institute (RPMI) 1640 medium supplemented with 10% heat-inactivated fetal bovine serum (FBS) and 1% Penicillin-Streptomycin (PS) at 37 °C, 5% CO_2_.

### Cell proliferation

To assess cell proliferation, 5 × 10^4^ cells of the indicated cells were seeded in 6-well plate. For the tetracycline inducible shRNA expressions, doxycycline was added to the culture at a final concentration of 3 μM. Trypan blue dye exclusion assays were performed every other day.

### Apoptosis assay

Apoptotic cells were detected by Annexin V Apoptosis Detection Kit APC (eBioscience Inc.). In brief, approximately 8 × 10^5^ cells of the indicated control and experimental groups were washed in phosphate-buffered saline (PBS), suspended in annexin-binding buffer, and then mixed with 5 μL of annexin V. The reaction mixtures were incubated for 30 min. After incubation, cells were diluted, stained with DAPI and processed for flow cytometric analysis.

### Real-time quantitative PCR (qRT-PCR)

Total RNA was isolated with RNeasy mini kit (Qiagen) and reverse transcribed with Reverse script kit (TOYOBO) to generate cDNA. Real-time PCR System (Applied Biosystems) according to the manufacturer’s instructions. The results were normalized to *GAPDH* levels. Relative expression levels were calculated using the 2^−ΔΔCt^ method. Primers used for qRT-PCR were listed in Supplementary Table 1.

### ChIP-qPCR

Chromatin immunoprecipitation assay (ChIP) was performed using SimpleChIP® Plus Enzymatic Chromatin IP Kit (Cell Signaling Technology, USA) according to the manufacturer’s instructions. In brief, cells were cross-linked in 1% formaldehyde in PBS for 10 min at room temperature. After glycine quenching, cell pellets were collected, lysed and then subjected to sonication with Q55 sonicator system (QSONICA, USA). The supernatant was diluted with the same sonication buffer, and processed for immunoprecipitation with anti-RUNX1 antibody (ab23980, abcam) at 4 °C overnight. The beads were then washed and DNA was reverse cross-linked and purified. Following ChIP, DNA was quantified by qPCR using the standard procedures for 7500 Real-Time PCR System (Applied Biosystems). The following primers were used for ChIP-qPCR; F 5′-ACTTTAGAGAGAAGGTAGCAT-3′ and R 5′-AGCTGTATCTCTCACTCAAAA-3′.

### siRNA interference

Specific shRNAs targeting human *RUNX1*, *SOS1* and *HER2* were designed and sub-cloned into pENTR4-H1tetOx1 and CS-RfA-ETV vectors (RIKEN BRC). Non-targeting control shRNA was designed against luciferase (sh_*Luc*). The target sequences were provided in Supplementary Table 2.

### Expression plasmids

We have amplified cDNAs for human *RUNX1* and *SOS1*, and then inserted them into CSIV-TRE-RfA-UbC-KT expression vector. All of the PCR products were verified by DNA sequencing.

### Production and transduction of lentivirus

For the production of lentivirus, HEK293T cells were transiently co-transfected with lentivirus vectors such as psPAX2 and pMD2.G by polyethylenimine (PEI, Sigma-Aldrich). Forty-eight hours after transfection, viral supernatants were collected and immediately used for infection, and then successfully transduced cells were sorted by flow cytometer Aria III (BD Biosciences).

### Luciferase reporter assay

Putative region of *SOS1* (−1000 bp to +200 bp of transcription start site) was cloned from the genomic DNA of K562 cells using the following primers; F 5′-CTCCCTGCAGAAATCCCGAG-3′ and R 5′-TCTTGGGACAAAACAAAACACCT-3′, and then subcloned into pGL4.20 [luc2/Puro] vector (Promega). Both pGL4.20 *SOS1* promoter vector and pRL-CMV control vector (TOYOBO B-Net Co., LTD.) were co-transfected into HEK293T cells that are stably-expressing shRNA of sh_*Luc* or expression vector of *RUNX1*. Promoter activities were measured using PicaGene Dual Sea Pansy Luminescence Kit (TOYOBO B-Net Co., LTD.) and detected by ARVO X5 (Perkin Elmer) according to the manufacturer’s instructions.

### Immunoblot

Immunoblotting was conducted as previously reported^30^. Briefly, cells were washed twice in ice-cold PBS and lysed in lysis buffer [50 mM Tris (pH 7.4), 100 mM NaCl, 0.1 mM EDTA, 1 mM phenylmethylsulfonyl fluoride, 1 mM Na_3_VO_4_, 1× protease inhibitor (Roche) and PhosSTOP (Roche)]. Whole cell extracts were separated by SDS-polyacrylamide gel electrophoresis (SDS-PAGE) and electro-transferred onto polyvinylidene difluoride membranes. Membranes were probed with the following primary antibodies: anti-RUNX1 (sc-365644, Santa Cruz Biotechnology, Inc.), anti-phosphorylated HER2 (#2243, Cell Signaling Technology), anti-HER2 (#2165, Cell Signaling Technology), anti-SOS1 (sc-10803, Santa Cruz Biotechnology, Inc.), anti-GAPDH (sc-47724, Santa Cruz Biotechnology, Inc.). For secondary antibodies, HRP-conjugated anti-rabbit IgG (#7074, Cell Signaling Technology) and anti-mouse IgG (#7076, Cell Signaling Technology) were used. Blots were visualized using Chemi-Lumi One Super (nacalai tesque, Inc.) and ChemiDoc^TM^ XRS + Imager (Bio-Rad Laboratories, Inc.) according to the manufacturer’s recommendations. We used total protein from human adult normal tissue (#P1234248, Biochain Inst., Inc.) as a normal control of stomach in Fig. 2c.

### Immunohistochemistry

Immunohistochemistry (IHC) was performed on formalin-fixed paraffin-embedded tissue sections using antibodies directed against human SOS1 antigen (ab140621, abcam) for xenograft experiments. The antigen-antibody complexes were visualized with Histofine Simple Stain MAX PO (Nichirei Biosciences). The tissue section images were captured using BZ-X700 All-in-One Fluorescence Microscope (Keyence, Japan).

### Immunoprecipitation

Cells were washed twice in ice-cold phosphate-buffered saline (PBS) and lysed in lysis buffer [50 mM Tris (pH 7.4), 100 mM NaCl, 0.1 mM EDTA, 1 mM phenylmethylsulfonyl fluoride, 1 mM Na_3_VO_4_, 1× protease inhibitor (Roche) and PhosSTOP (Roche)]. Whole cell extracts were processed for immunoprecipitation with the following antibodies at 4 °C overnight. Anti-SOS1 antibody (sc-10803, Santa Cruz Biotechnology, Inc.), anti-HER2 antibody (#2165, Cell Signaling Technology) and anti-Normal Rabbit IgG antibody (#2729, Cell Signaling Technology). Protein G Sepharose^TM^ 4 Fast Flow (17-0618-01, GE Healthcare) was added and an hour after incubation at 4 °C, the beads were washed three times in ice-cold wash buffer [20 mM Tris-HCl, 0.15 M NaCl, 0.1% Tween 20]. Samples were separated by SDS-polyacrylamide gel electrophoresis (SDS-PAGE) and electro-transferred onto polyvinylidene difluoride membranes. Membranes were probed with the following primary antibodies: anti-HER2 (#2165, Cell Signaling Technology) and anti-SOS1 (sc-10803, Santa Cruz Biotechnology, Inc.). For secondary antibodies, HRP-conjugated anti-rabbit IgG (#7074, Cell Signaling Technology) was used. Blots were visualized using Chemi-Lumi One Super (nacalai tesque, Inc.) and ChemiDoc^TM^ XRS + Imager (Bio-Rad Laboratories, Inc.) according to the manufacturer’s recommendations.

### Human phospho-RTK array

Human phospho-RTK array was performed using Human Phospho-RTK Array Kit (R&D Systems, Inc.) according to the manufacturer’s instructions. In brief, cells were solubilized in Lysis Buffer 17 prepared with protease inhibitors. After blocking with Array Buffer 1 for an hour, the arrays were incubated with the protein lysate at 4 °C overnight, and then washed and incubated with Anti-Phospho-Tyrosine HRP Detection Antibody in Array Buffer 2 for 2 hours at room temperature. Detection was by Chemi Reagent Mix and signals were captured using ChemiDoc^TM^ XRS + Imager (Bio-Rad Laboratories, Inc.) according to the manufacturer’s recommendations.

### Statistics

Statistical significance of differences between control and experimental groups was assessed by a two-tailed unpaired Student’s t-test, and was declared if the p value was less than 0.05. Equality of variances in two populations was calculated with F-test. The results were represented as the average ± SEM values obtained from three independent experiments. In transplantation experiments, animals were randomly allocated to each experimental group, and the treatments were given with blinding.

### Mice

NOD/Shi-scid IL-2RγKO (NOG) mice were purchased from Central Institute of Experimental Animals, Japan. Littermates were used as controls in all experiments.

### Xenograft mouse model

Xenograft mouse model of human cancer cell lines were developed using NOG mice. For gastric cancer models, 2 × 10^6^ cells/body of MKN45 cells were injected subcutaneously at the right dorsal flank. Seven days after injection, mice were treated with PI polyamides (320 μg/kg body weight, twice a week IV injections) or with the equivalent amount of dimethyl sulfoxide (DMSO).

### Synthesis of PI polyamides

Synthesis of Chb-M’ was conducted as previously reported^22^. Briefly, Py-Im polyamide supported by oxime resin was prepared in a stepwise reaction by Fmoc solid-phase protocol. The product with oxime resin was cleaved with *N*,*N*-dimethyl-1,3-propane diamine (1.0 mL) at 45 °C for 3 hours. The residue was dissolved in the minimum amount of dichloromethane and washed with dimethyl ether to yield a 59.6 mg. To the crude compound (59.6 mg, 48.1 μmol), a solution of chlorambucil (32.6 mg, 107 μmol), benzotriazole-1-yl-oxy-tris-pyrrolidino-phosphonium hexafluorophosphate (PyBOP) (101 mg, 195 μmol) and *N*,*N*-diisopropylethylamine (100 μL, 581 μmol) in *N*,*N*-dimethylformamide (DMF) (300 μL) was added. The reaction mixture was incubated for 1.5 hours at room temperature, washed with dimethyl ether and DMF for three times and dried in vacuo. The crude product was purified by reversed-phase flash column chromatography (water with 0.1% trifluoroacetic acid/MeCN). After lyophilization, product was obtained (30.2 mg, 19.8 μmol). Machine-assisted polyamide syntheses were performed on a PSSM-8 (Shimadzu) system with computer-assisted operation. Flash column purifications were performed by a CombiFlash Rf (Teledyne Isco, Inc.) with C18 RediSep Rf Flash Column. Electrospray ionization time-of-flight mass spectrometer by using positive ionization mode and proton nuclear magnetic resonance (^1^H NMR) spectra were recorded with a JEOL JNM ECA-600 spectrometer operating at 600 MHz and in parts per million (ppm) downfield relative to tetramethylsilane used as an internal standard to verify the quality of synthesized PI polyamides.

### Study approval

All animal studies were properly conducted in accordance with the Regulation on Animal Experimentation at Kyoto University, based on International Guiding Principles for Biomedical Research Involving Animals. All procedures employed in this study were approved by Kyoto University Animal Experimentation Committee (Permit Number: Med Kyo 14332).

## Electronic supplementary material

Supplemental Data
