# Supplementary material for: RUNX1 positively regulates the ErbB2/HER2 signaling pathway through modulating SOS1 expression in gastric cancer cells
Source: Sci Rep. 2018 Apr 23;8:6423. doi: 10.1038/s41598-018-24969-w (PMC5913281; doi:10.1038/s41598-018-24969-w)

**Supplementary Information**

**RUNX1 positively regulates the ErbB2/HER2 signaling pathway through modulating SOS1 expression in gastric cancer cells.**

Yoshihide Mitsuda^1^, Ken Morita^1^, Gengo Kashiwazaki^2^, Junichi Taniguchi^2^, Toshikazu Bando^2^, Masahiro Hirata^3^, Moeka Obara^1^, Tatsuki R Kataoka^3^, Manabu Muto^4^, Yasufumi Kaneda^5^, Tatsutoshi Nakahata^6^, Pu Paul Liu^7^, Souichi Adachi^1,8^, Hiroshi Sugiyama^2^ and Yasuhiko Kamikubo^1^.

^1^Department of Human Health Sciences, Graduate School of Medicine, Kyoto University, Sakyo-ku, Kyoto 606-8507, Japan

^2^Department of Chemistry, Graduate School of Science, Kyoto University, Sakyo-ku, Kyoto 606-8502, Japan

^3^Department of Diagnostic Pathology, Kyoto University Hospital, Sakyo-ku, Kyoto 606-8507, Japan

^4^Department of Therapeutic Oncology, Graduate School of Medicine, Kyoto University, Sakyo-ku, Kyoto 606-8507, Japan

^5^Division of Gene Therapy Science, Department of Genome Biology, Graduate School of Medicine, Osaka University, Osaka 565-0871, Japan

^6^Drug Discovery Technology Development Office, Center for iPS cell research and application (CiRA), Kyoto University, Sakyo-ku, Kyoto 606-8507, Japan

^7^Oncogenesis and Development Section, National Human Genome Research Institute, National Institutes of Health, Bethesda, MD, 20892, USA

^8^Department of Pediatrics, Graduate School of Medicine, Kyoto University, Sakyo-ku, Kyoto 606-8507, Japan

**
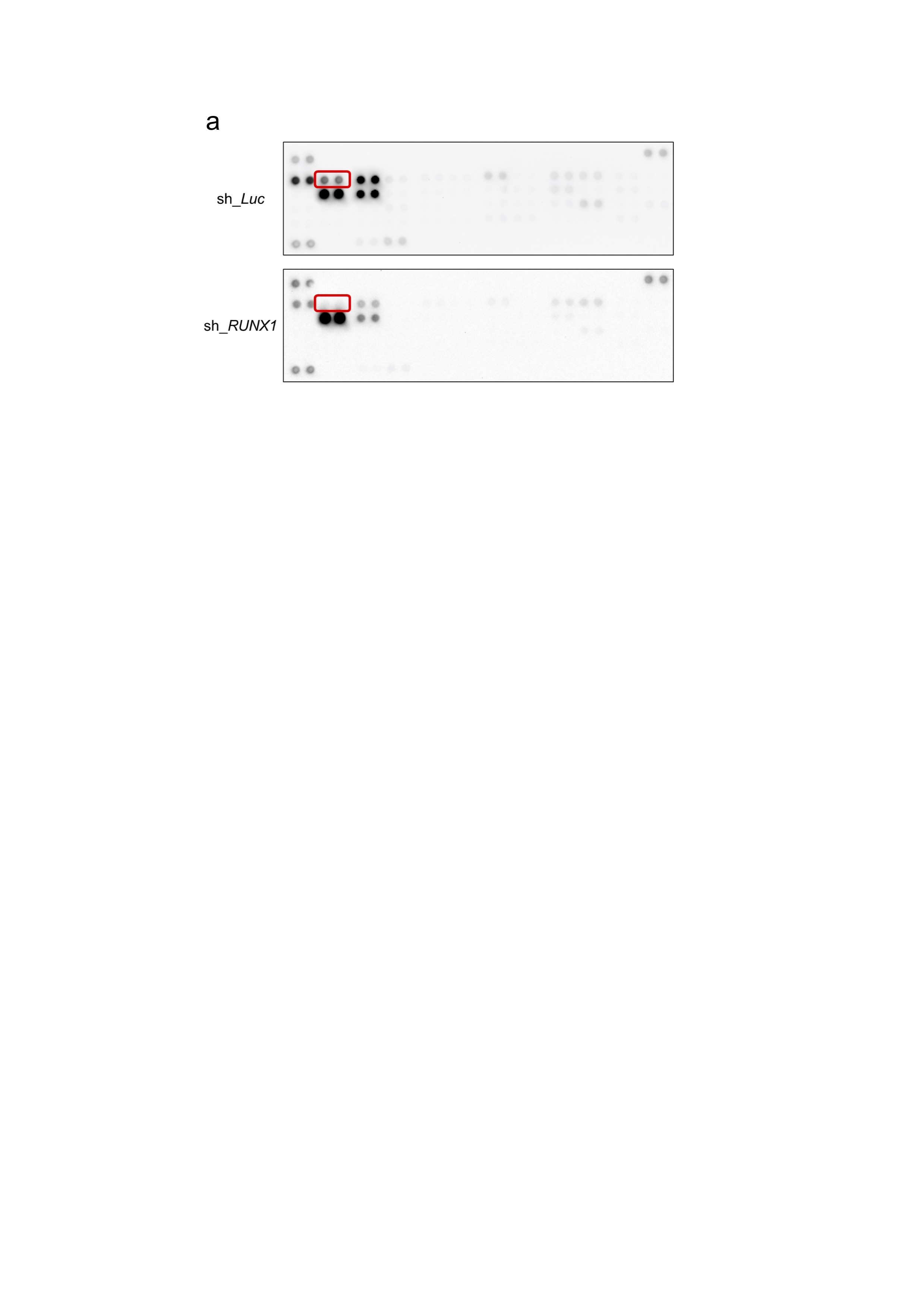
**

**Supplementary Figure 1**

(a) Phosphorylation status of RTKs by sh_*RUNX1*. Each receptor was spotted in duplicates. Red ellipse indicates spots of HER2.


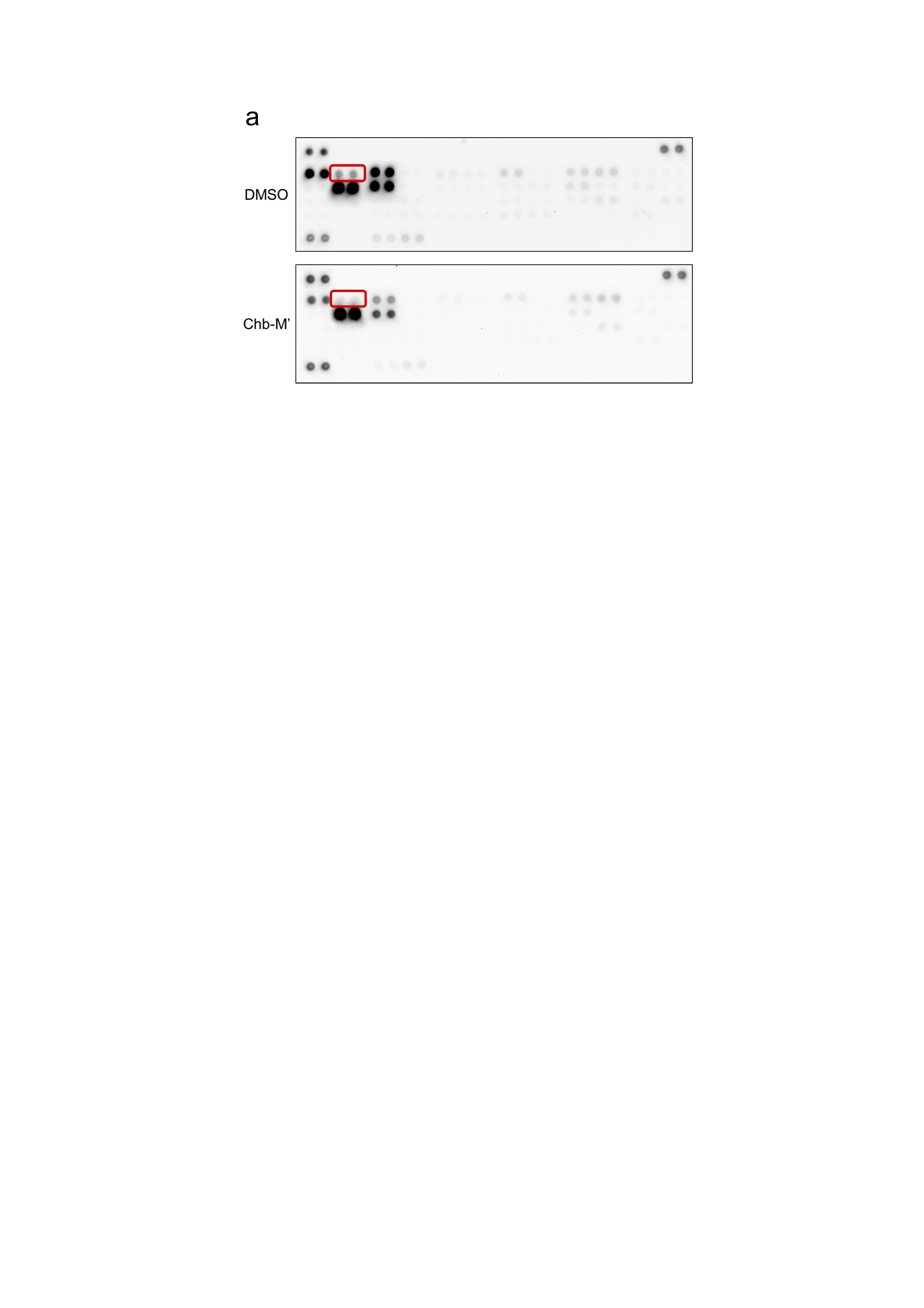


**Supplementary Figure 2**

(a) Phosphorylation status of RTKs in MKN45 cells treated with DMSO or 1 μM Chb-M’ for 48 hours. Each receptor was spotted in duplicates. Red ellipse indicates spots of HER2.


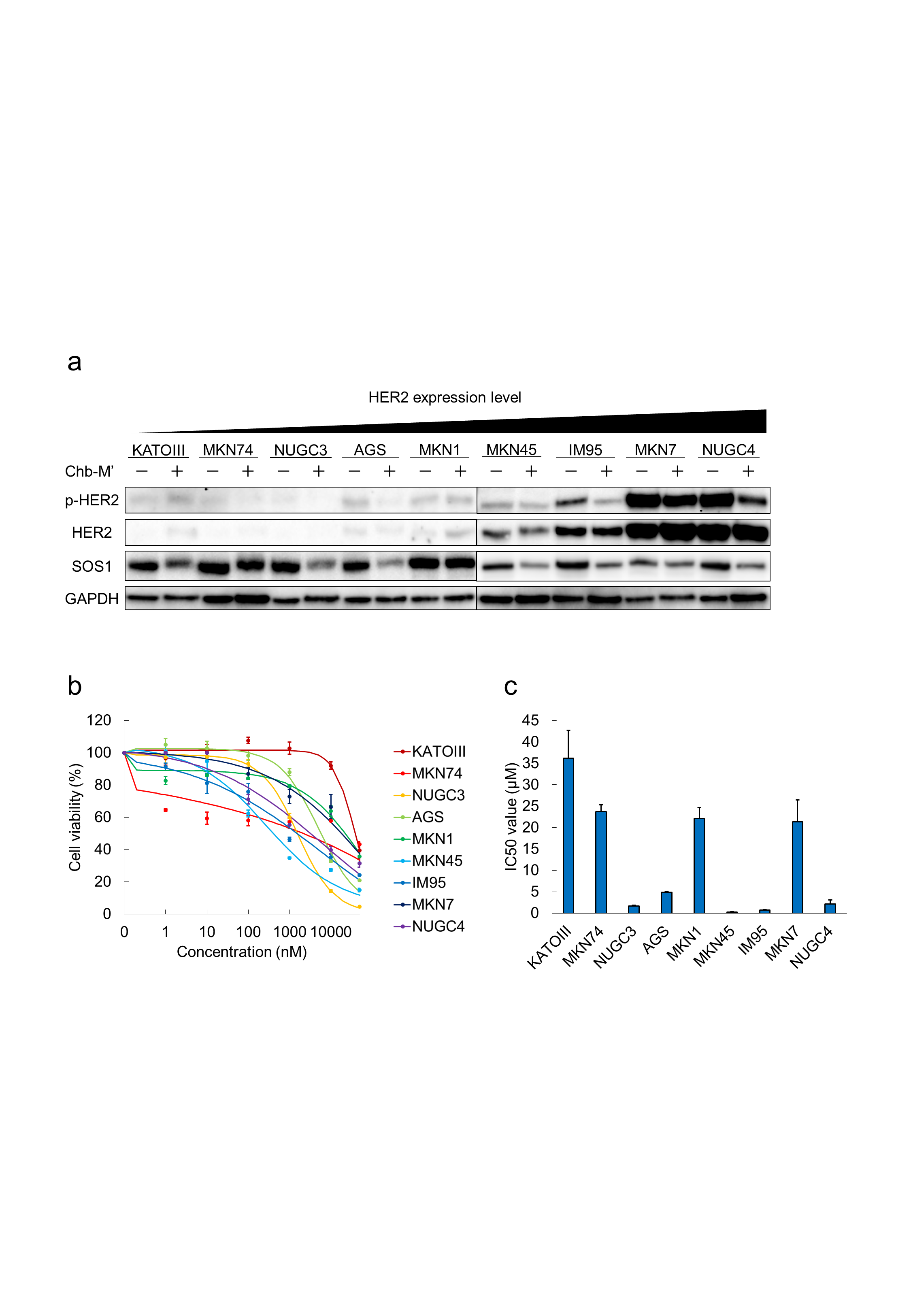


**Supplementary Figure 3**

(a) Efficacy of Chb-M’ against 9 gastric cancer cells. Cells were treated with DMSO or 10 μM Chb-M’. Seventy-two hours after treatment, cell lysates were processed for immunoblotting.

(b) Dose-response curves of Chb-M’ in gastric cancer cells. Cells were treated with the indicated concentration of Chb-M’. Seventy-two hours after treatment, cell viability was examined by WST assay (n=3).

(c) IC50 values of Chb-M’ against gastric cancer cells. Cells were treated with various concentrations of Chb-M’ for 72 hours (n=3).

**
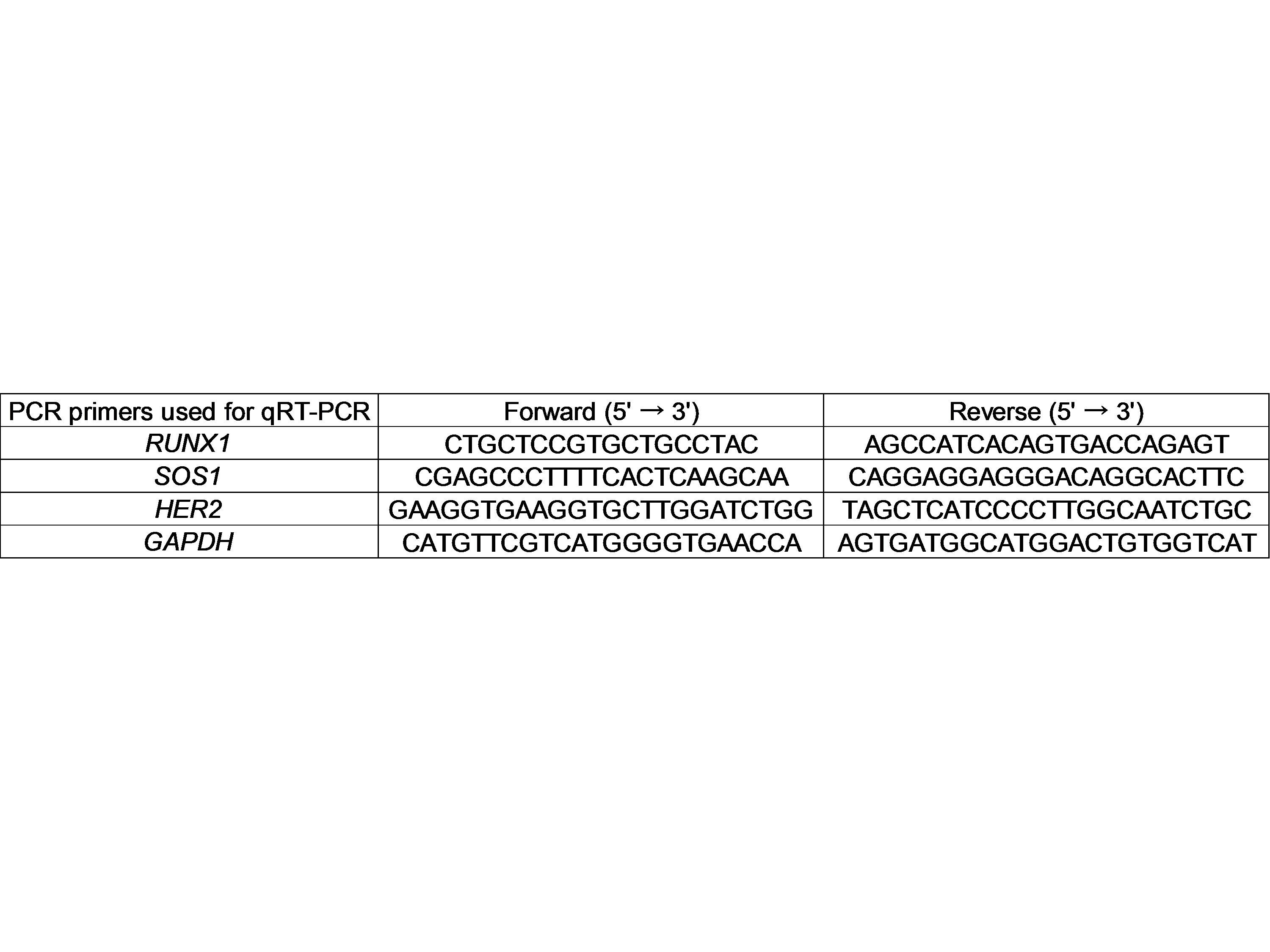
**

**Supplementary Table 1**

List of primers used for RT-qPCR experiments in this study.


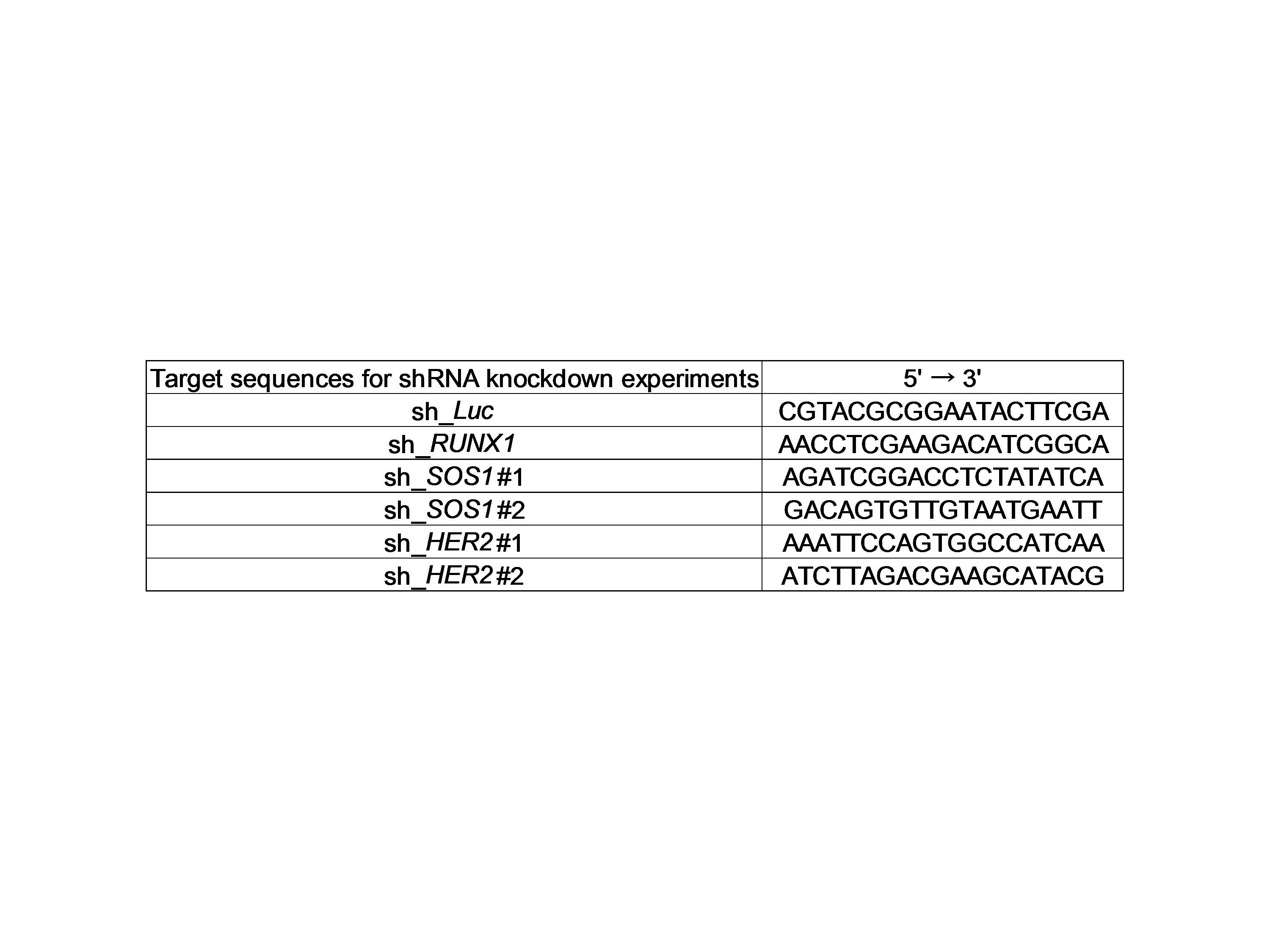


**Supplementary Table 2**

List of target sequences for shRNA-mediated knockdown experiments in this study.

**Unedited Gels**

**Fig. 1e**
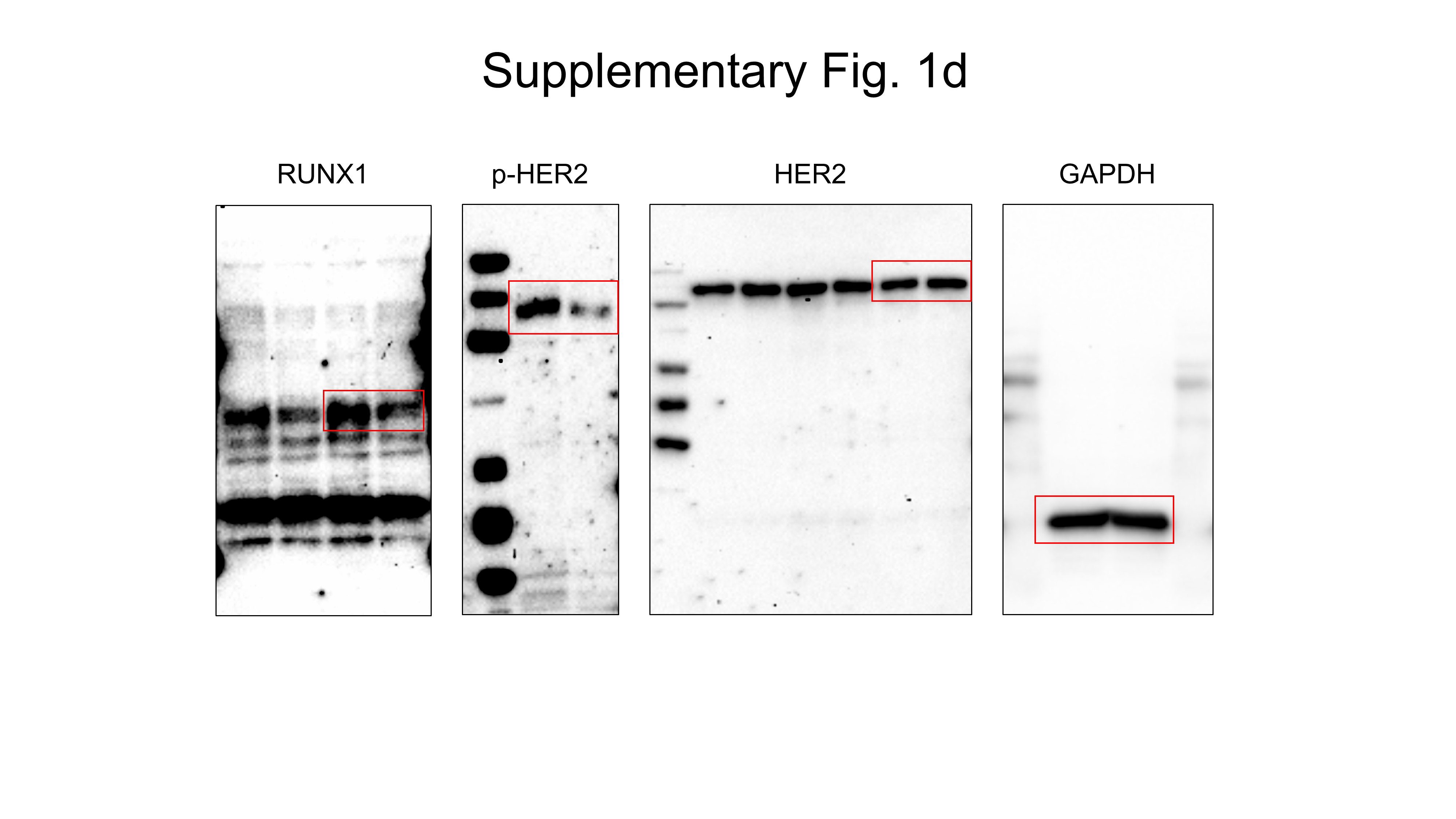


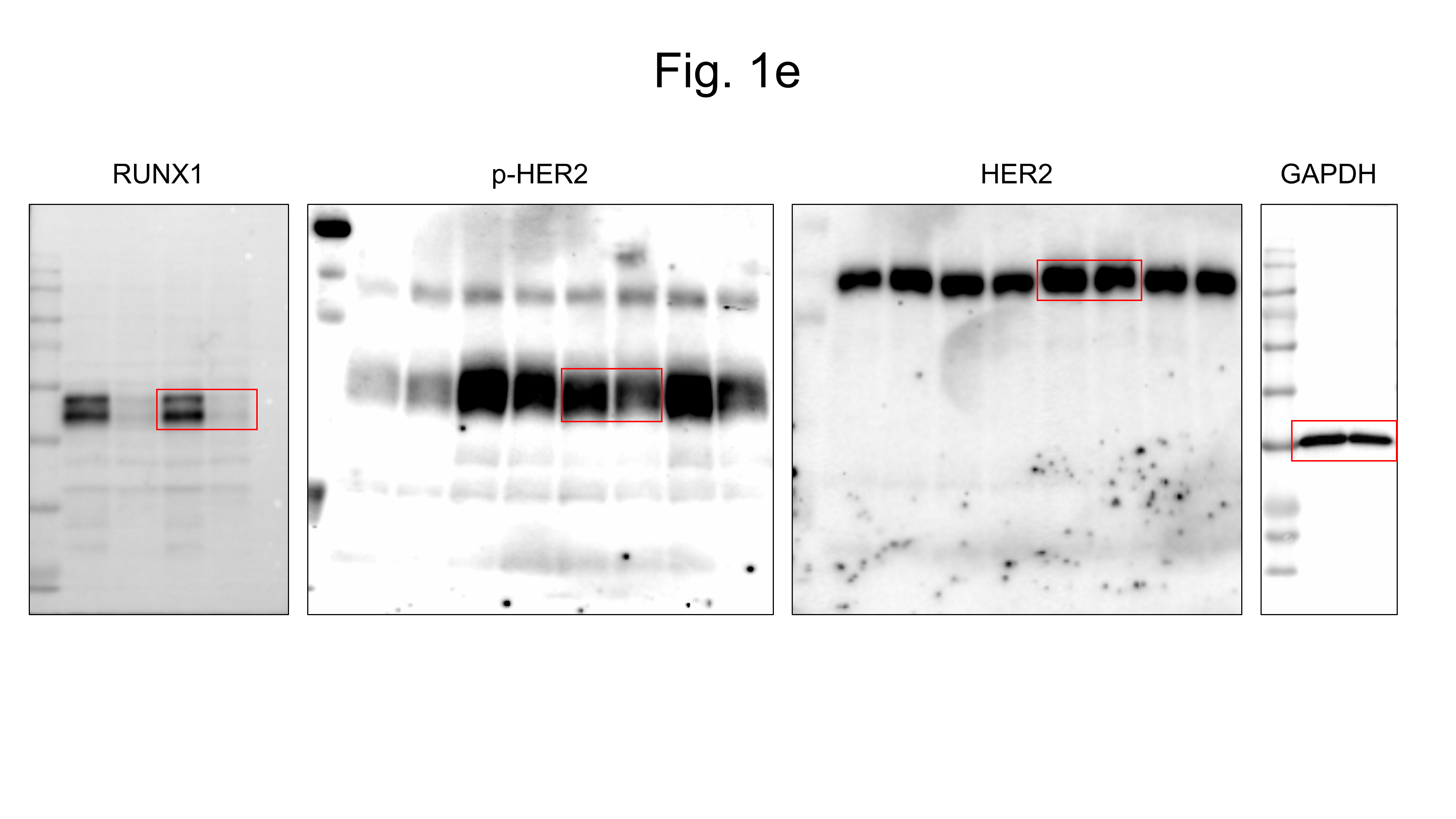


**Fig. 2c**


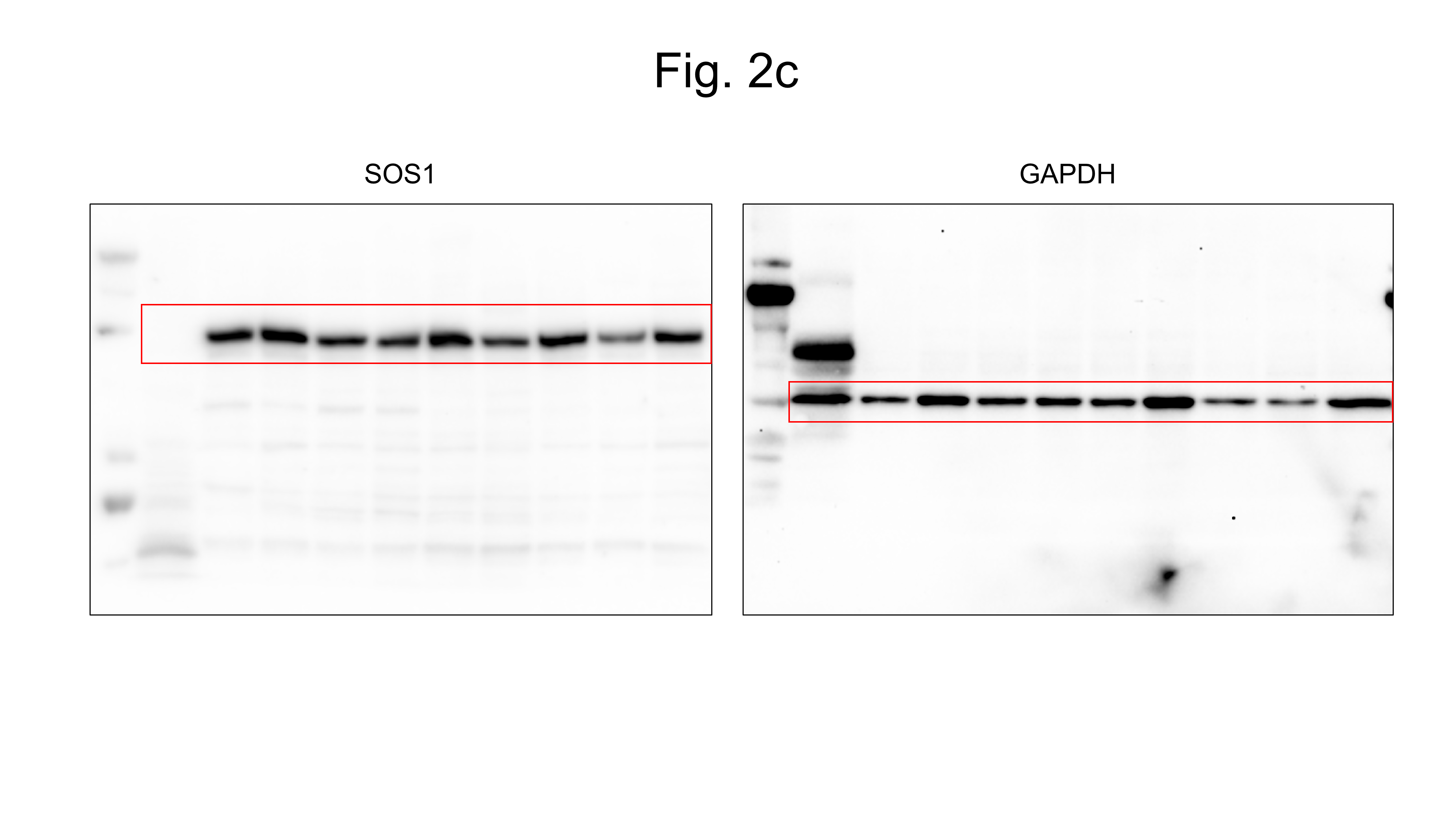


**Fig. 2e**


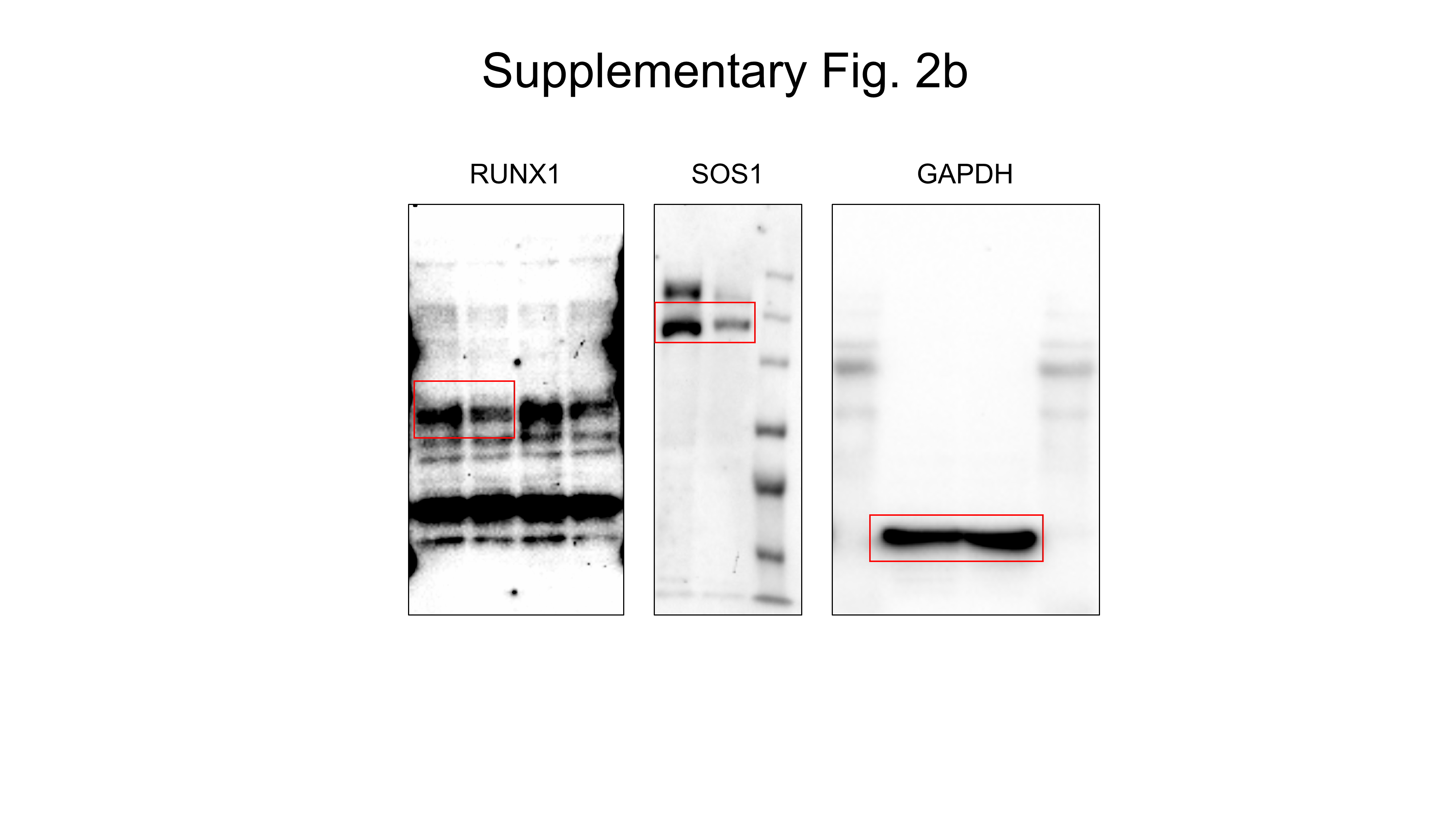


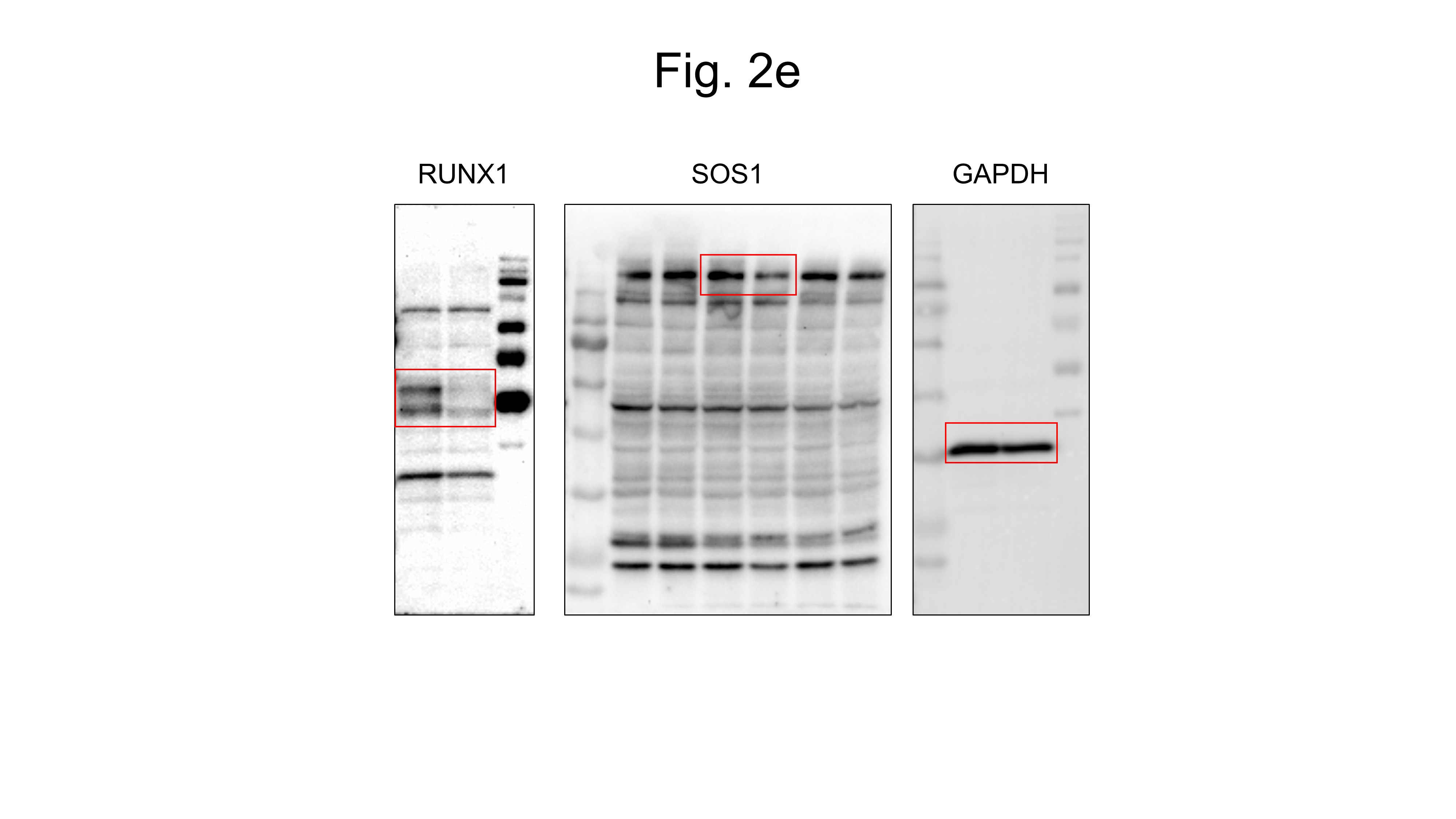


**Fig. 3b**


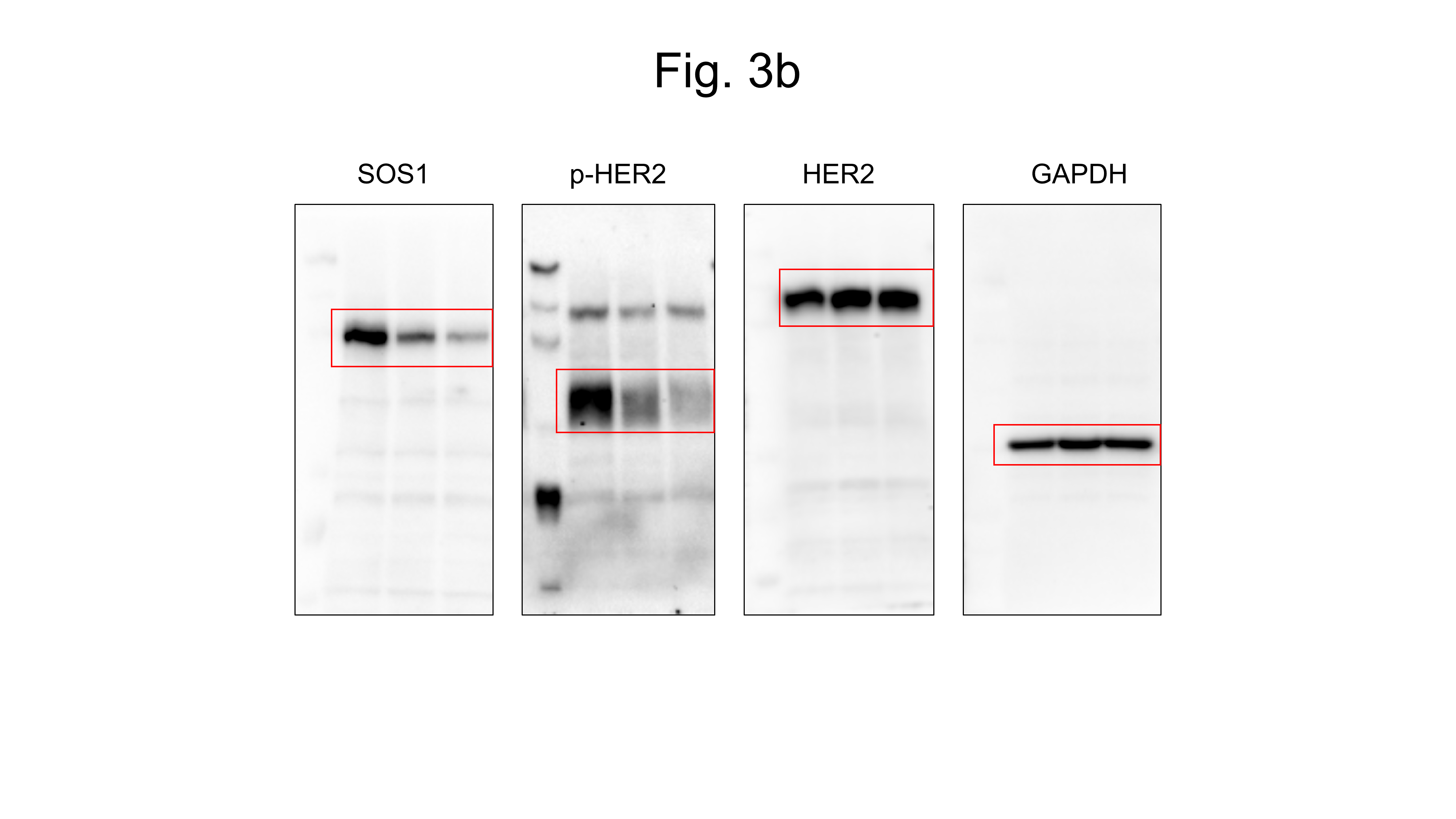


**Fig. 3e**


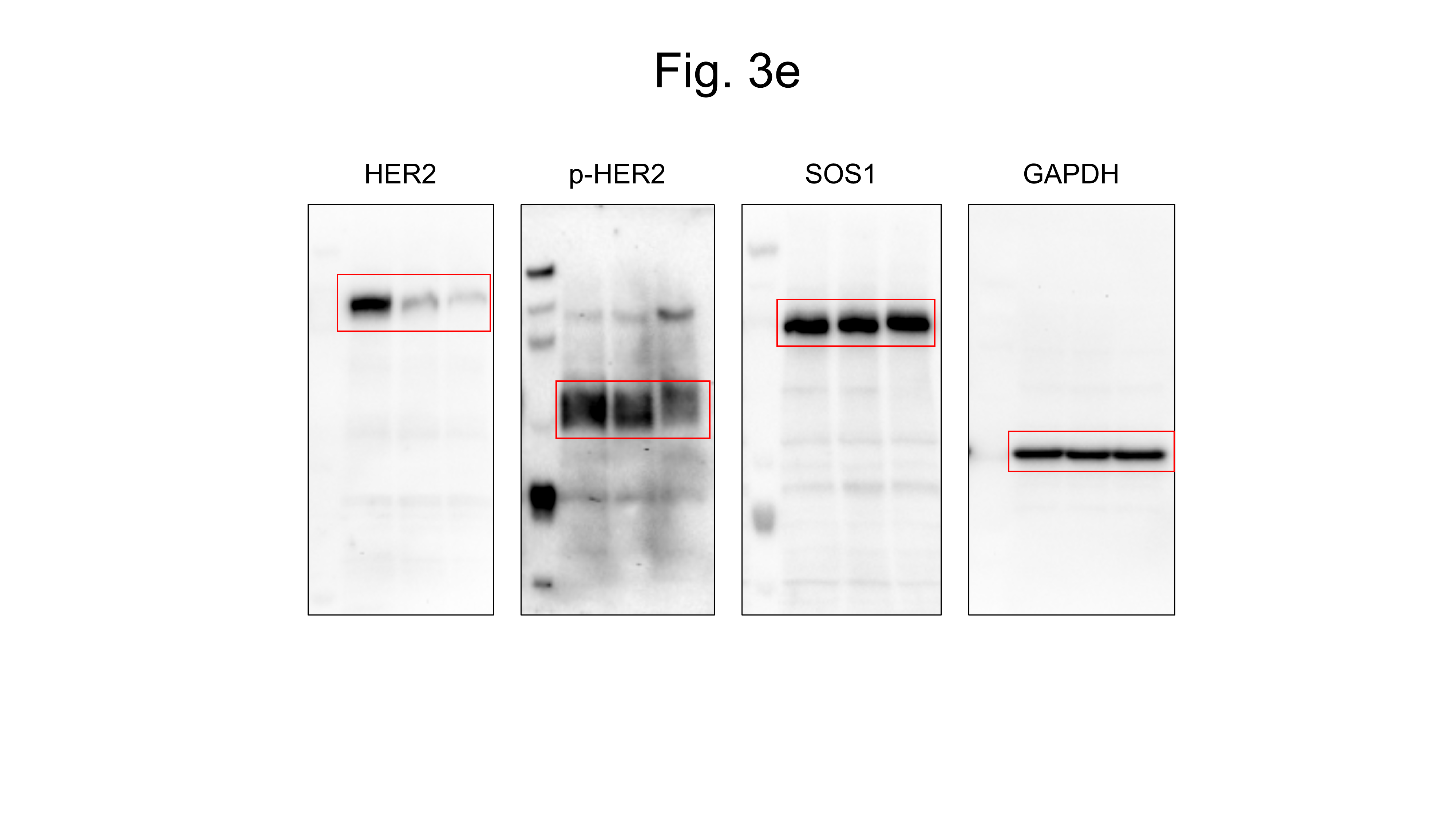


**Fig. 3g**


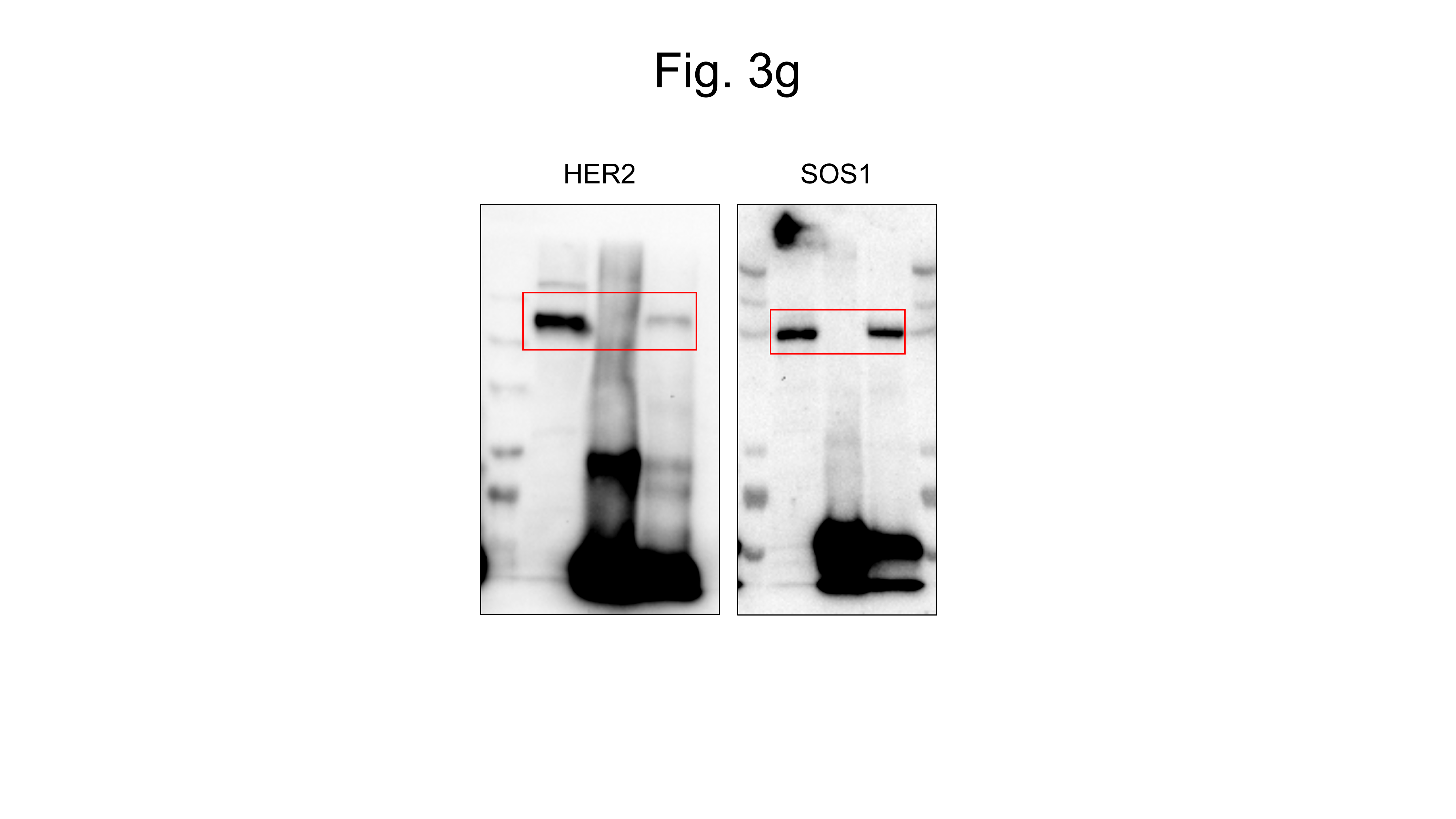


**Fig. 3h**
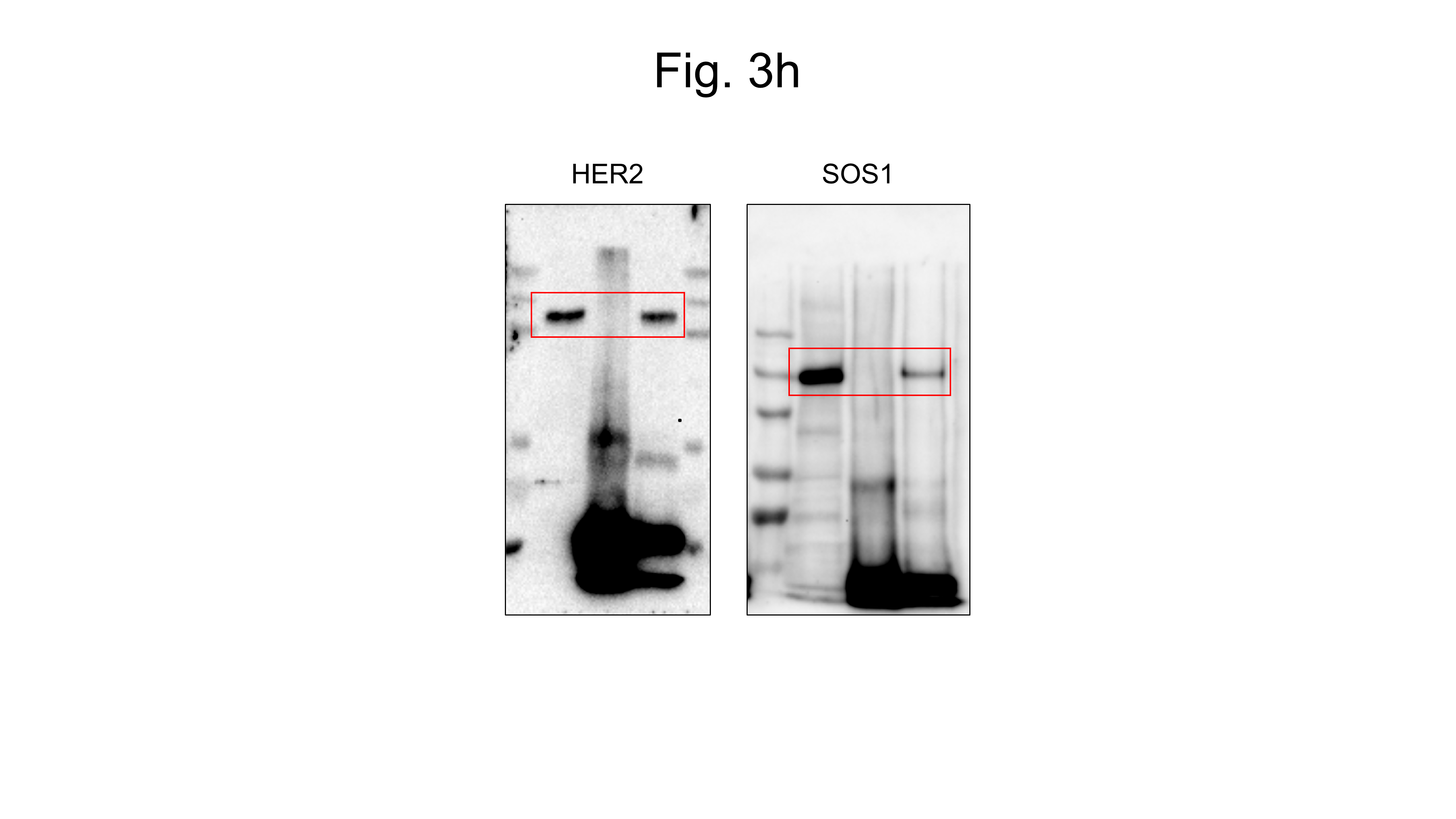


**Fig. 5a**


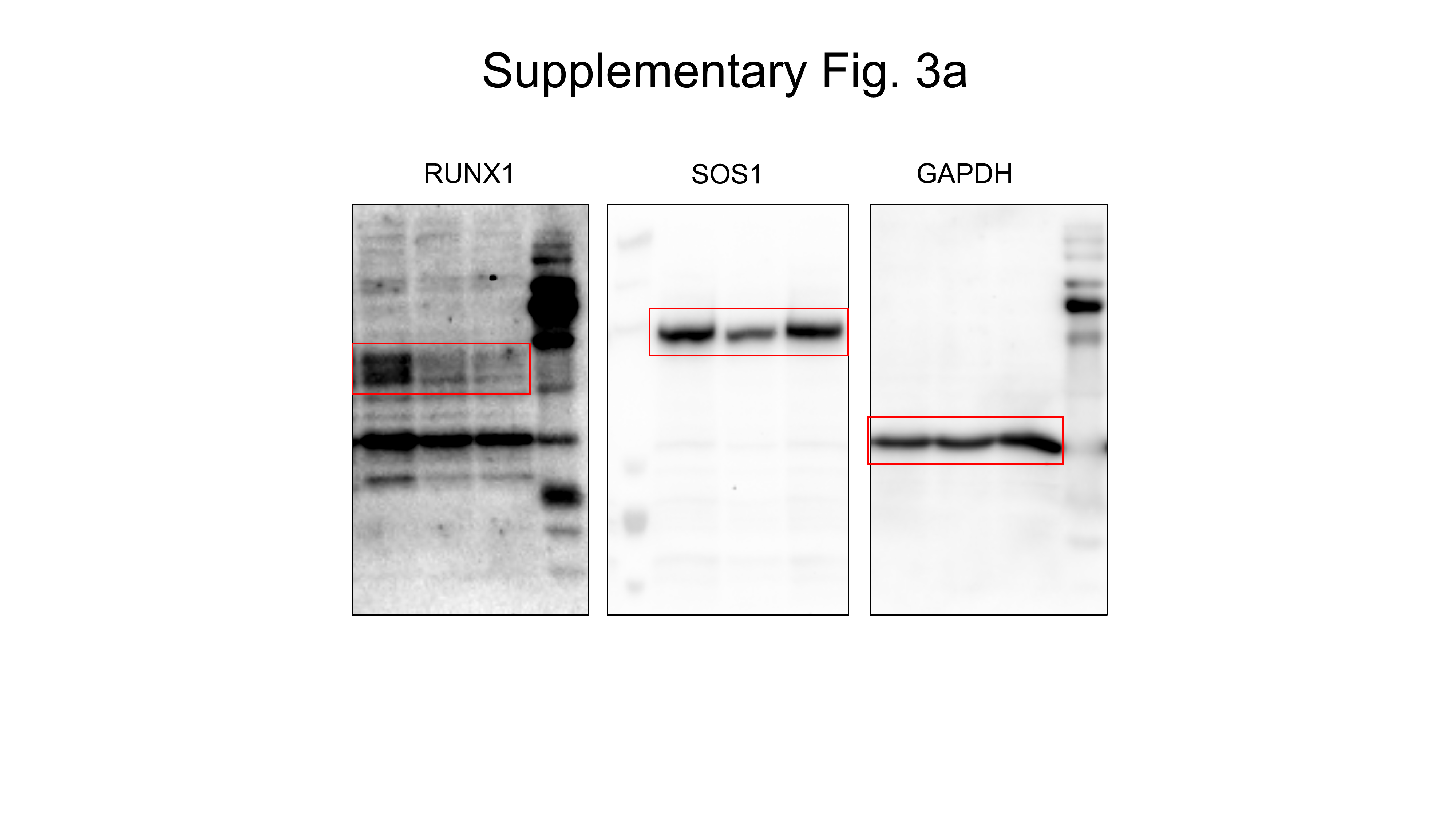


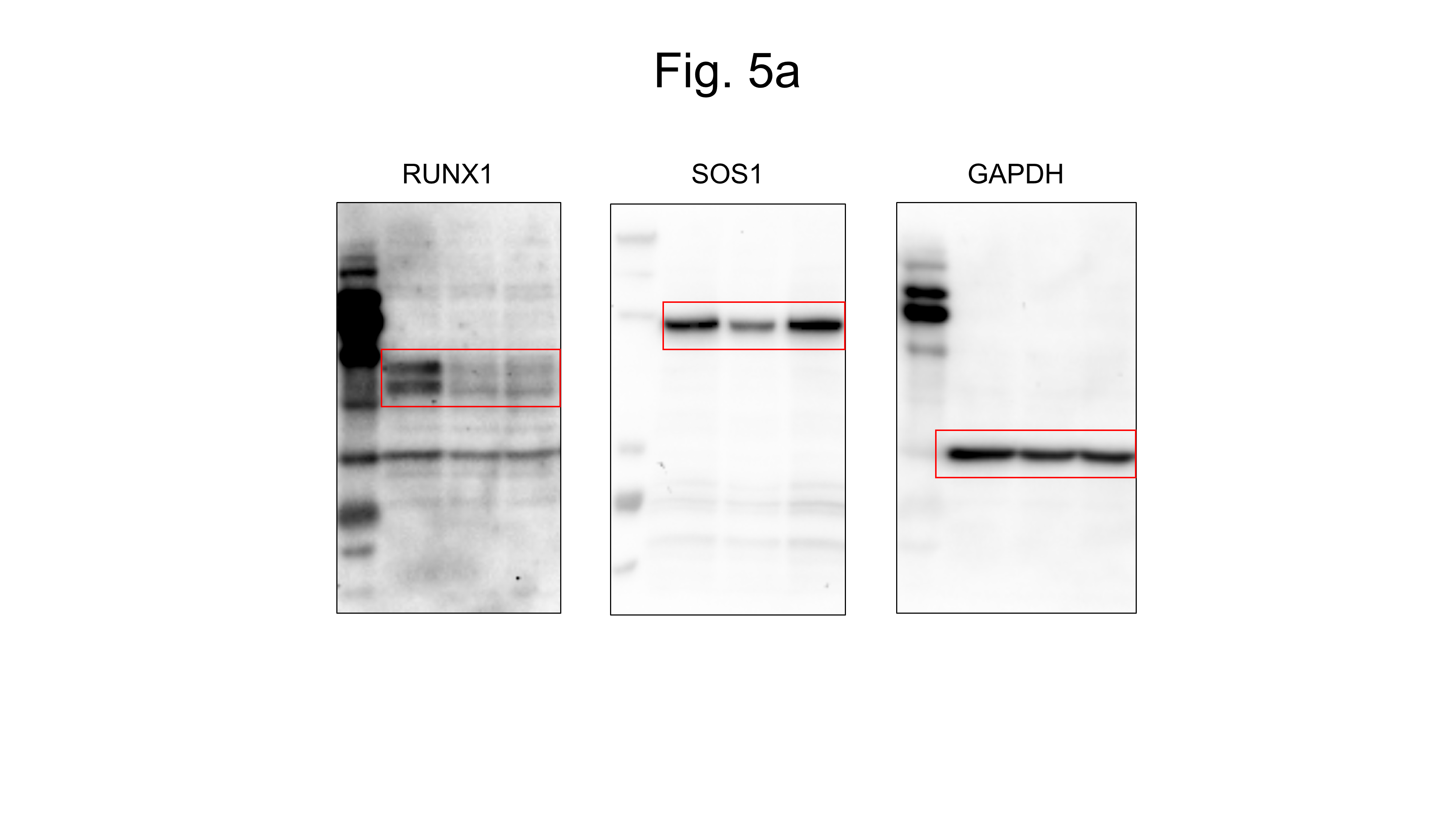


**Fig. 6c**


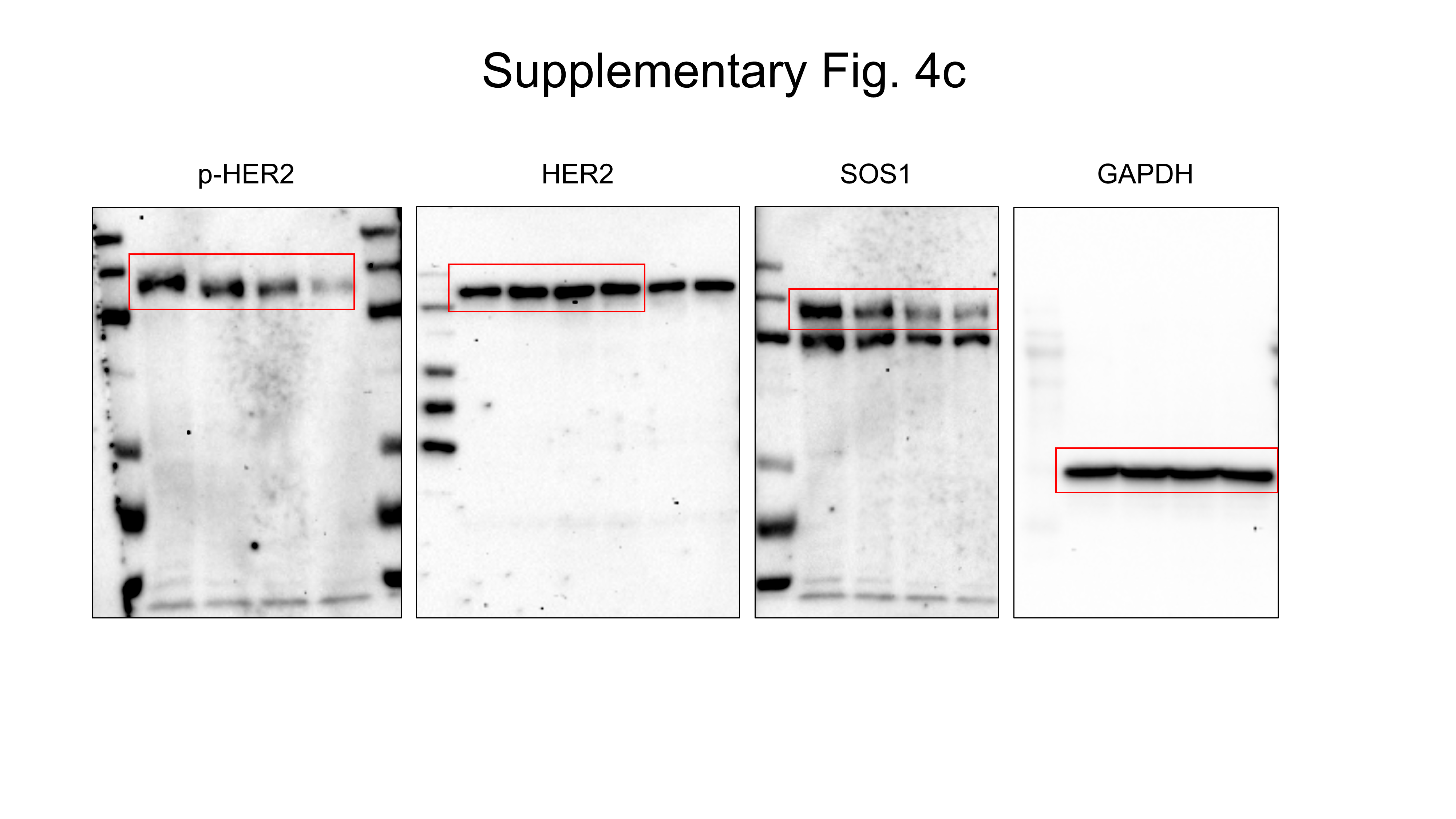


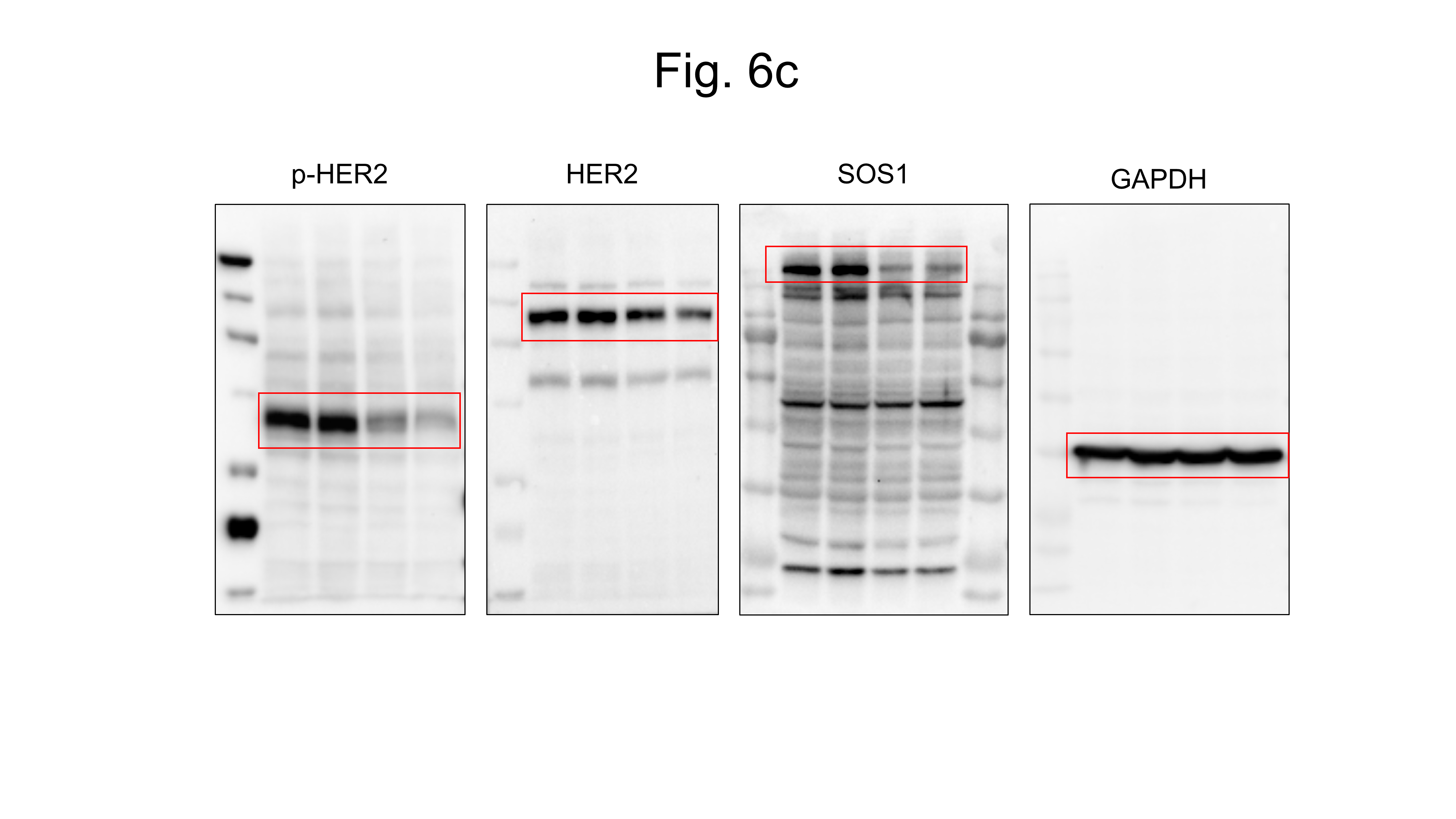


**Fig. 8a**
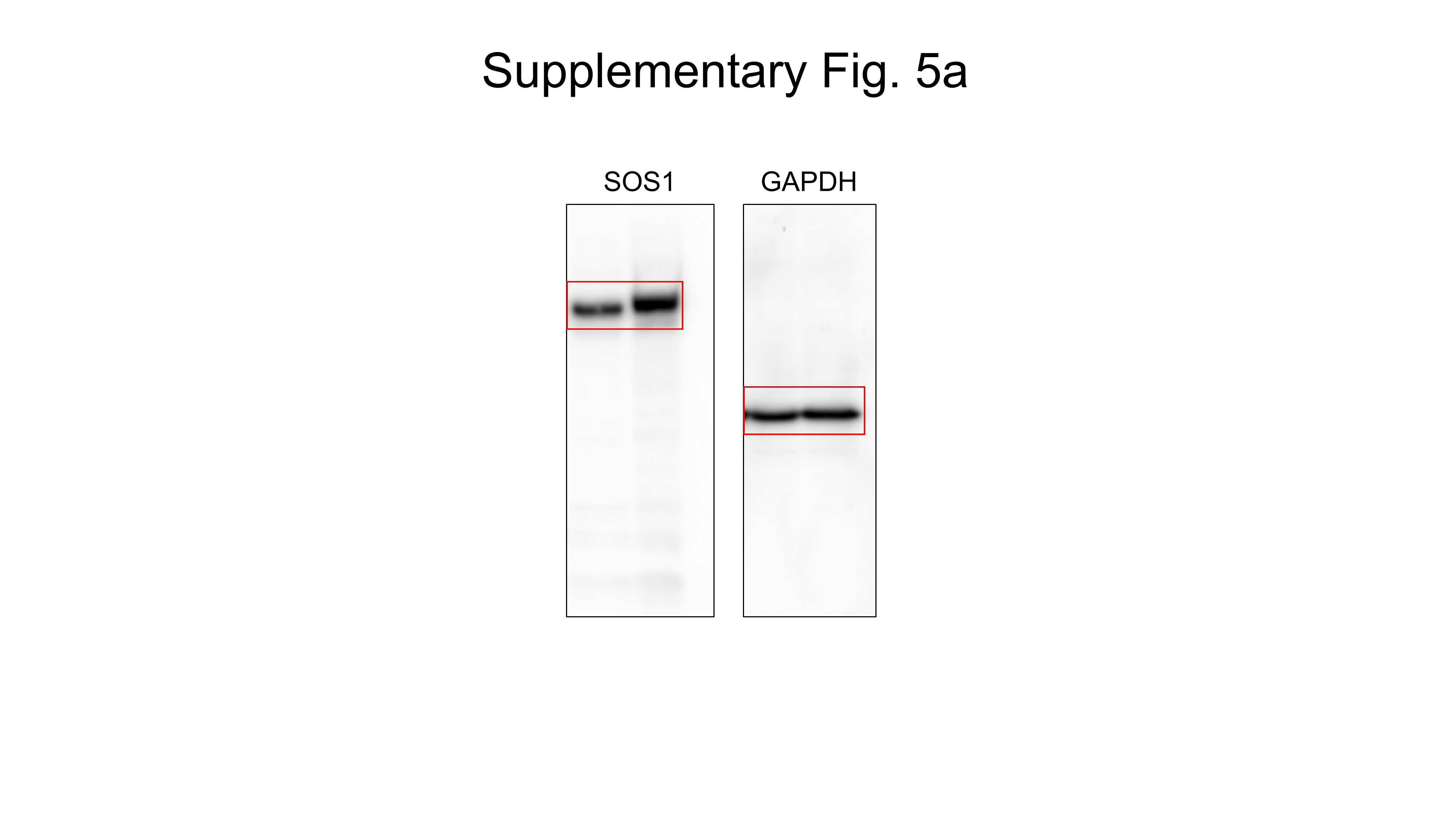

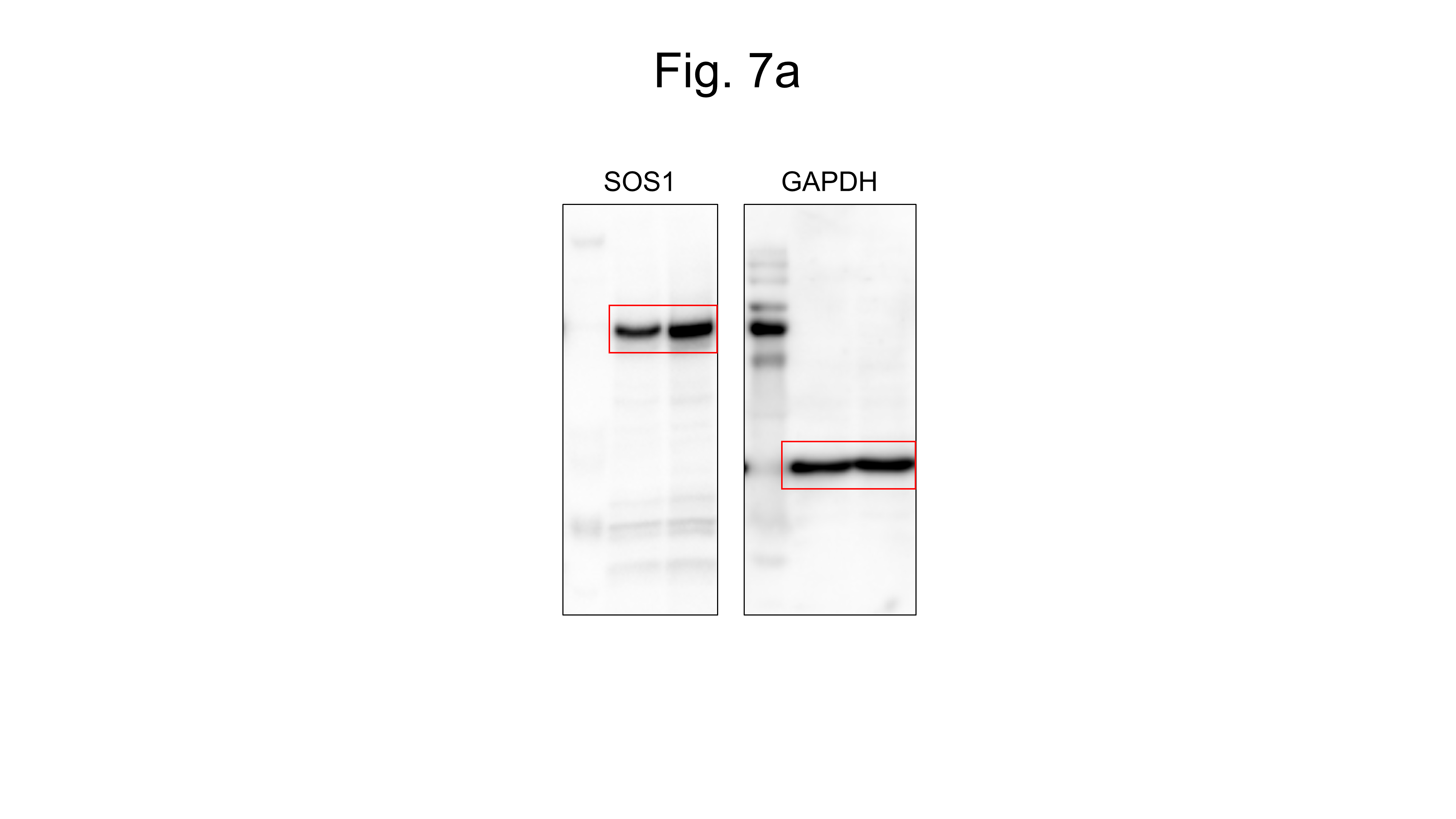


**Supplementary Fig. 3a**
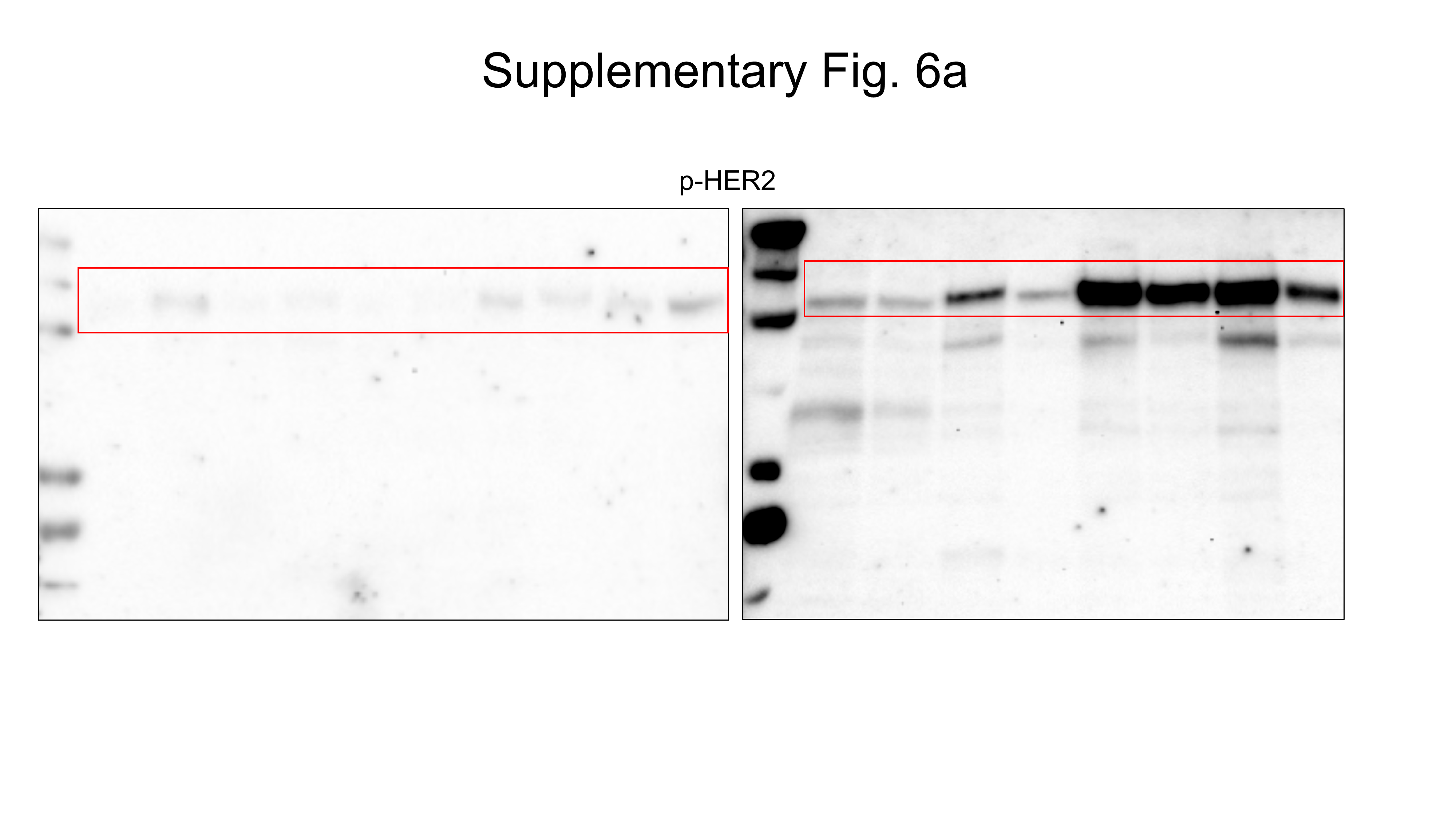

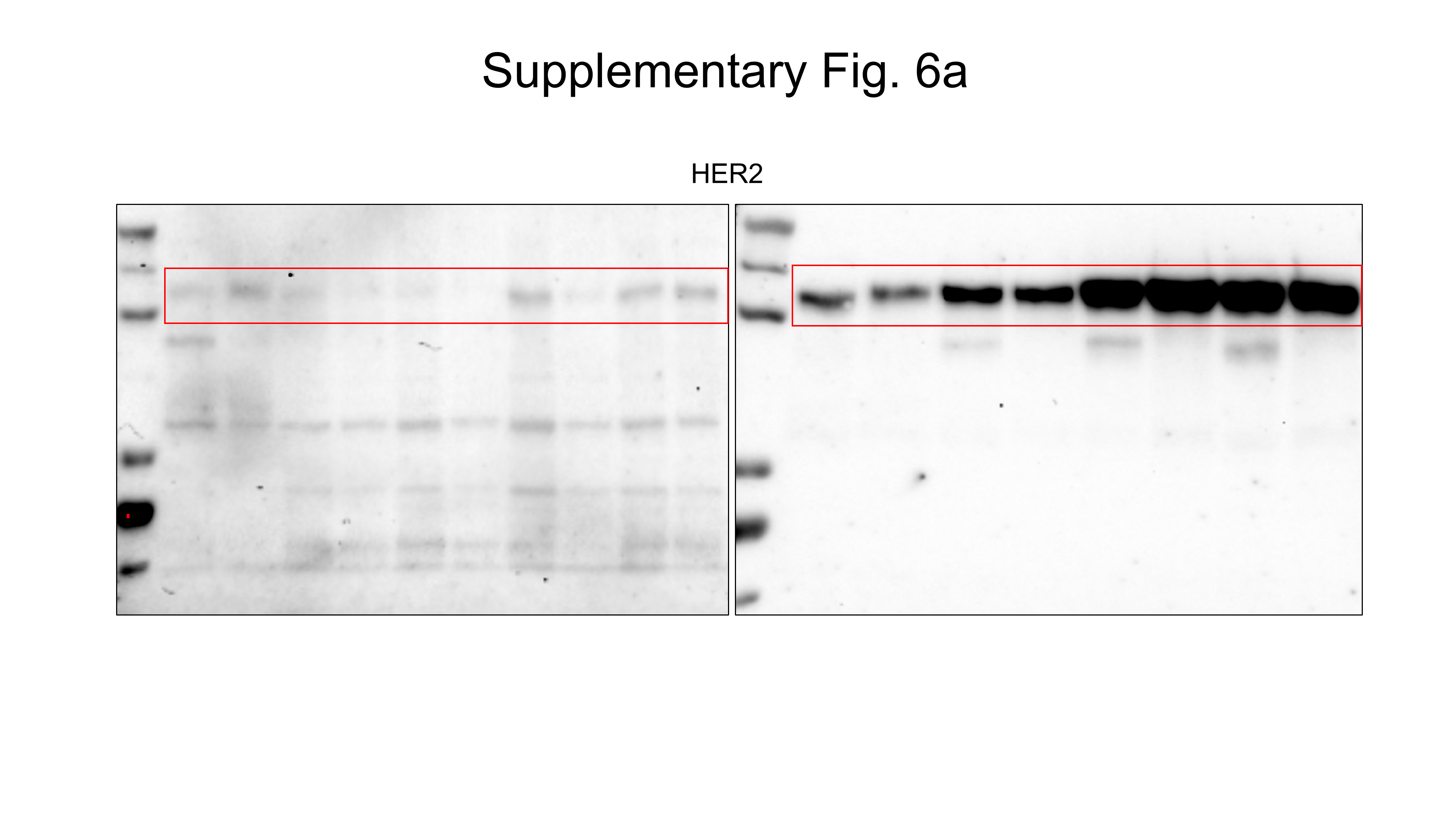

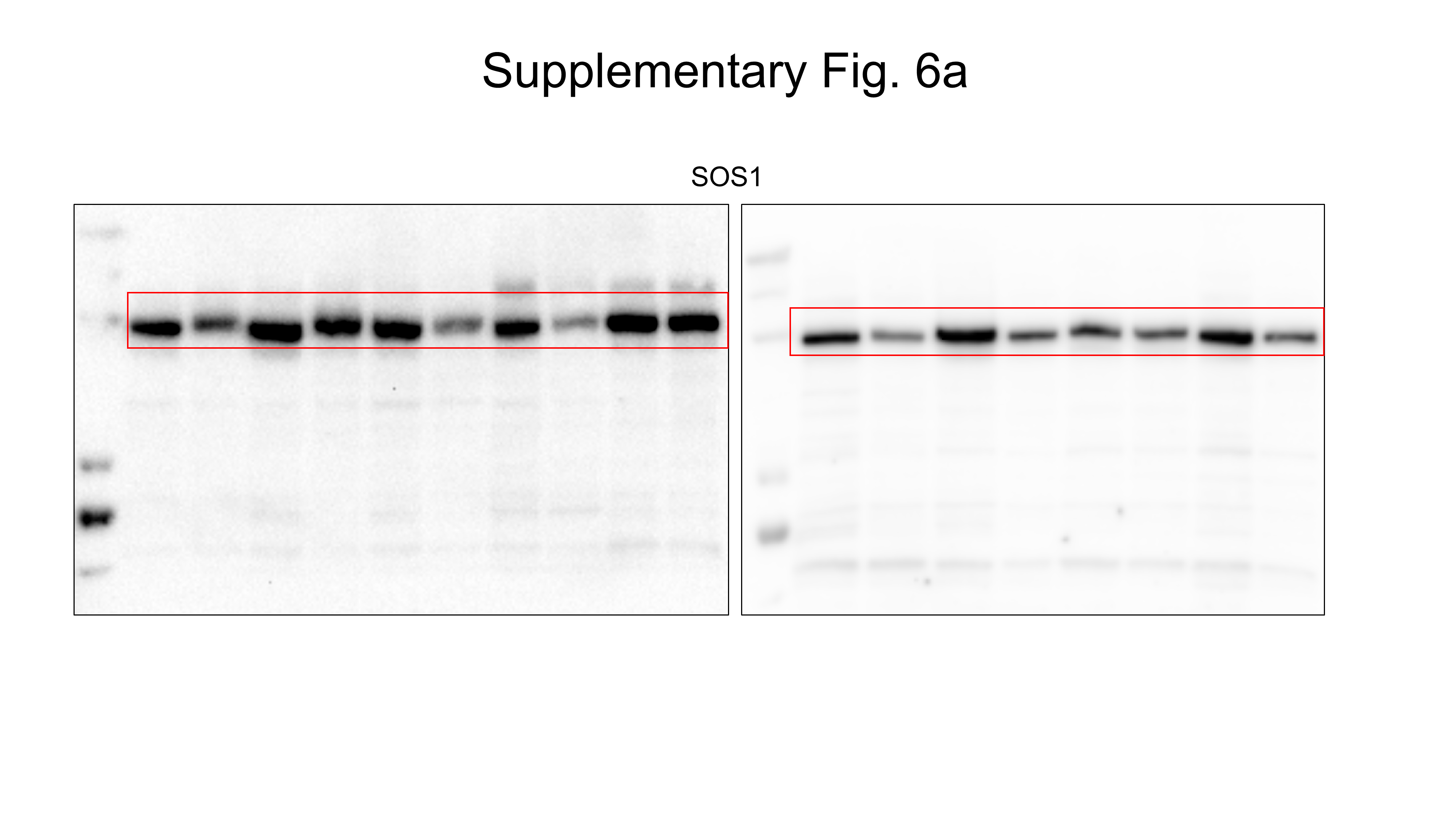

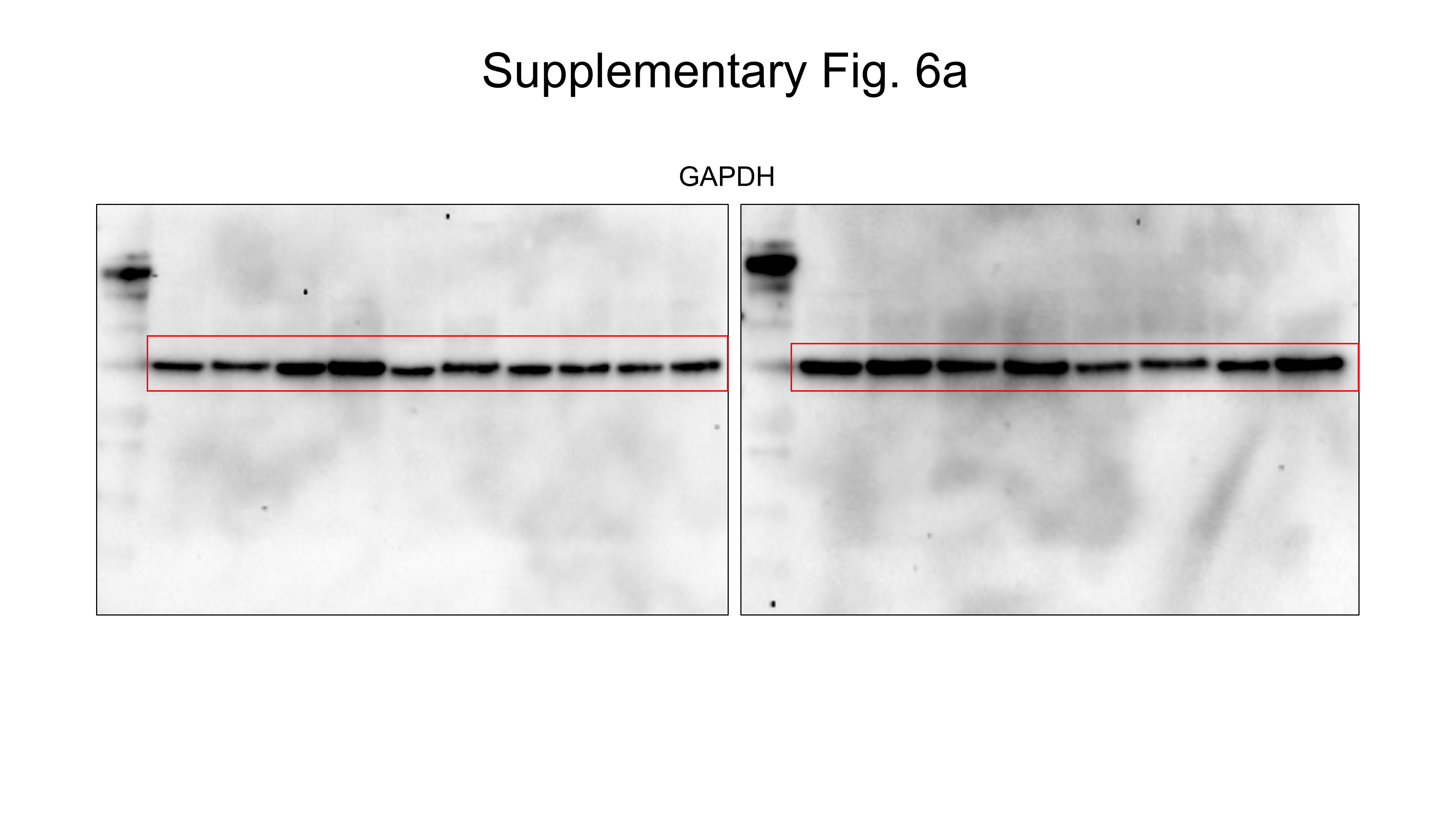

Supplement: Supplementary file 1 — Supplemental Data [file 41598_2018_24969_MOESM1_ESM.docx]
